# Orai1 acts as a novel Ca^2+^ signal switch, balancing erythropoiesis through KLF1 regulation

**DOI:** 10.1038/s12276-026-01651-0

**Published:** 2026-03-04

**Authors:** Yoon Young Lee, Hyebin Koh, Jieun Kim, Sun-Uk Kim, Jong-Hee Lee, Chan Young Park

**Affiliations:** 1 Department of Biological Sciences, College of Information and Biotechnology, Ulsan National Institute of Science and Technology, Ulsan, Republic of Korea; 2 National Primate Research Center, Korea Research Institute of Bioscience and Biotechnology, Cheongju, Republic of Korea; 3 Department of Functional Genomics, KRIBB School of Bioscience, University of Science and Technology, Daejeon, Republic of Korea; 4 Futuristic Animal Resource & Research Center, Korea Research Institute of Bioscience and Biotechnology, Cheongju, Republic of Korea; 5 Department of Advanced Bioconvergence, KRIBB School of Bioscience, University of Science and Technology, Daejeon, Republic of Korea; 6 Department of Biomedical Engineering, College of Information and Biotechnology, Ulsan National Institute of Science and Technology, Ulsan, Republic of Korea

**Keywords:** Calcium signalling, Haematopoietic stem cells

## Abstract

Terminal erythropoiesis, the final stage of red blood cell maturation, is orchestrated by erythropoietin (EPO) and the master transcription factor, Kruppel-like factor 1 (KLF1). Recent studies highlight the importance of Ca^2+^ signaling in erythroid maturation; however, the underlying mechanisms remain elusive. Here we identify Orai1 as a novel EPO-responsive Ca^2+^ channel in erythroid cells, serving as a dynamic regulatory toggle that modulates KLF1 transcription and facilitates distinct phases of erythroid maturation. During the early stages, EPO-activated Orai1 suppresses KLF1 transcription through Ca^2+^-dependent NFAT2 activation and promoter binding, pausing erythroid maturation. As maturation progresses, Orai1 expression decreases, transitioning KLF1 regulation to an EPO–STAT5 pathway, thereby maintaining KLF1 expression and promoting terminal erythropoiesis. Using HUDEP-2 cells, umbilical cord blood and human pluripotent stem cell-derived CD71⁺ erythroblasts, we observed a progressive downregulation of Orai1 and reduction in intracellular Ca^2+^ levels during terminal maturation. The functional inactivation of Orai1 via R91W mutants and CRISPR–Cas9 knockout enhanced KLF1 expression, leading to increased erythroid-specific gene expression, accelerated erythroid maturation, higher levels of globin production and improved enucleation efficiency. This study unveils the EPO–Orai1–Ca^2+^–NFAT2–KLF1 axis as a critical regulatory checkpoint in erythropoiesis and highlights Orai1 downregulation as a potential strategy to enhance clinical red blood cell production by promoting erythrocyte maturation.

## Ca2+ signaling and Orai1 drive terminal erythropoiesis dynamics

This study explores how red blood cells (RBCs) mature, focusing on a protein called Orai1. RBCs are crucial for carrying oxygen in the body, and understanding their development can help improve blood-related treatments. The researchers identified a gap in knowledge about how Orai1 affects RBC maturation. Researchers found that Orai1 plays a key role in controlling Ca^2+^ signals during RBC development. They used human stem cells and other cell models to investigate this process. By manipulating Orai1, they observed changes in the expression of KLF1, a protein important for RBC maturation. Researchers discovered that reducing Orai1 activity enhances RBC maturation by increasing KLF1 levels and improving the production of β-globin, a component of hemoglobin. This suggests that Orai1 acts as a checkpoint in RBC development.

This summary was initially drafted using artificial intelligence, then revised and fact-checked by the author.

## Introduction

Terminal erythropoiesis, the final stage of red blood cell (RBC) maturation, involves the highly specialized differentiation processes of CD71^+^ erythroid-committed precursors into mature erythrocytes. This process is tightly regulated by erythropoietin (EPO), which activates a network of signaling molecules, including Grb2, PI3K, PLC, JAK2 and STAT5, to control the expression of key erythroid genes^1–3^. EPO-mediated signaling pathways promote the proliferation and survival of erythroblasts, thereby enhancing RBC production^4–6^. Conversely, EPO also impedes erythroid maturation by suppressing hemoglobin maturation and enucleation^7–9^. These dual roles underscore the necessity of precise regulation of EPO signaling during terminal erythropoiesis to ensure balanced RBC production.

Erythroid maturation relies on the tightly controlled expression of Kruppel-like factor 1 (KLF1), a master transcription factor critical for the transcription of erythroid-specific genes, particularly those involved in hemoglobin transition, and enucleation^10–12^. Moreover, the exogenous overexpression of KLF1 augments the terminal maturation of RBCs, resulting in higher enucleation efficiency^13,14^. Given its pivotal role, the precise regulation of KLF1 expression is of considerable interest during the final stages of erythropoiesis. EPO signaling positively regulates TAL1^15,16^, a key activator of KLF1 transcription, particularly during late erythropoiesis^17,18^. In addition, the JAK2–STAT5 pathway, a canonical downstream signaling route of EPO, has been shown to increase DDX5 expression^19^, a critical component of the KLF1 enhancer^20^. Despite these advances, the mechanisms through which EPO signaling regulates KLF1 in a stage-specific manner expression to drive terminal erythroid maturation remains incompletely understood.

An increasing body of research has explored the involvement of Ca^2+^ ions in transmitting EPO signals to KLF1 expression. EPO modulates Ca^2+^ influx through pathways dependent on signal transduction from EPO receptor (EPOR) to phospholipase C-γ1 (PLCγ1), leading to the activation of inositol 1,4,5-trisphosphate receptor (IP3R), which promotes Ca^2+^ depletion from the endoplasmic reticulum (ER) and triggers extracellular Ca^2+^ influx^21–23^. Moreover, NFAT2, a Ca^2+^-associated transcription factor, was reported to directly bind to the KLF1 promoter and regulate the KLF1 transcription in murine model^24^. However, the molecular mechanism underlying the EPO-mediated Ca^2+^ signals regulating KLF1 and terminal erythroid maturation has yet to be elucidated.

Orai is an essential Ca^2+^ channel for cellular differentiation, facilitating extracellular Ca^2+^ influx known as store-operated Ca^2+^ entry (SOCE)^25^. Upon Ca^2+^ depletion in ER, stromal interaction molecule (STIM) proteins oligomerize and activate the Orai channel. The involvement of Orai in the differentiation of various hematopoietic cells, including lymphoid (T cell^26^, B cell^27^ and NK cells^28^), granulocyte (neutrophil)^29^ and macrophage^30^, is well-documented. However, the specific function of Orai1 in erythroid maturation remains poorly understood. Previous studies have highlighted the functional relevance of Orai-STIM in erythroid differentiation. Patients with mutations in Orai1 or STIM1 exhibited hepatosplenomegaly^31,32^, raising a potential link to stress erythropoietic expansion. Moreover, STIM1/2-deficient leukemic mice show increased Ter119^+^ mature erythroblasts and mitigated leukemia-associated anemia^33^. Genetic polymorphisms in Orai1 have also been associated with the risk of EPO resistance, further underscoring its potential role in erythropoiesis^34^.

This study elucidates the critical role of Orai1 as a novel signal checkpoint in EPO signaling, linking external EPO cues to the KLF1 expression during terminal erythropoiesis. In CD71^+^ proerythroblasts, EPO activates Orai1-dependent Ca^2+^ signaling, leading to the NFAT2 dephosphorylation and nuclear translocation, which suppresses KLF1 gene transcription. As erythroid maturation progresses, Orai1 expression is downregulated, shifting EPO-mediated Ca^2+^ signaling toward STAT5, which sustains KLF1 expression. Disabling Orai1 effectively expedites erythrocyte development, enhancing KLF1 expression, β-globin production and enucleation across various cellular models, including immortalized HUDEP-2 cells and human pluripotent stem cell (hPS cell)-derived CD71^+^ erythroblasts. Overall, our findings contribute to a deeper understanding of the developmental process of erythrocytes, shed light on the role of Orai1–Ca^2+^ signaling in terminal erythropoiesis and offer valuable insights for in vitro strategies for generating mature RBCs.

## Materials and methods

### Generation and validation of *Orai1*^−/−^-KO human iPS cell lines

To generate the *Orai1*^−/−^-knockout (KO) human induced pluripotent stem cells (iPS cells), we designed a plasmid on the basis of homology-directed repair. The Orai1-targeting vector contained an 800-b 5′ homology arm, EF1 promoter driven mCherry–IRES–puromycin resistance cassette and an 800-b 3′ homology arm. The homology arms were amplified from human genomic DNA by KOD One PCR Master Mix (TOYOBO). The mCherry sequence was cloned in frame immediately upstream of the Orai1 start codon. Vectors were linearized with SpeI for electroporation. A guide RNA (GTGGTGCTGCCGCCGCTTGG) was cloned info a CRISPR–Cas9 plasmid (Addgene) to induce a double-strand break. iPS cells were co-electroporated with the Orai1–mCherry–IRES–puromycin and CRISPR–Cas9 plasmids using P3 Primary Cell 4D-Nucleofectors X Kit and Nucleofector Device (Lonza). KO cells were selected with 1 μg/ml puromycin (Thermo Fisher Scientific), and antibiotic-resistant colonies were transferred to Matrigel-coated 24-well plates. Transfected cells were confirmed by fluorescence microscopy.

### RBC differentiation from hPS cells

Pluripotent cells (human iPS cells/human embryonic stem cells) were maintained on Matrigel (Corning)-coated plates in E8 medium (STEMCELL Technologies) and passaged weekly using ReLeSR (STEMCELL Technologies). For erythroid differentiation, colonies were dissociated into single cells with Accutase (STEMCELL Technologies). To make embryoid bodies, 3 × 10^4 ^cells were seeded per well in 96-well round-bottomed ultra-low attachment plates (Corning). Mesendodermal induction was performed for 5 days in albumin polyvinylalcohol essential lipids (APEL) medium containing 40 ng/ml BMP4, 50 ng/ml VEGF, 40 ng/ml stem cell factor (SCF) and 5 ng/ml bFGF, followed by five additional days with 30 ng/ml TPO and 50 ng/ml Flt3L. On day 10, eight embryoid bodies were transferred onto Matrigel-coated 12-well plates in APEL medium supplemented with 50 ng/ml VEGF, 40 ng/ml SCF, 5 ng/ml bFGF 50 ng/ml Flt3L, 30 ng/ml TPO, 30 ng/ml IL-3, 30 ng/ml IL-6 and 5 U/ml EPO.

### Western blots

Cells were washed with PBS and lysed in buffer (100 mM Tris–HCl, 150 mM NaCl and 1% Triton X-100) at 4 °C for 10 min. After centrifugation (13,000 rpm, 10 min, 4 °C), supernatants were mixed with 4× SDS dye (250 mM Tris–HCl, 400 mM DTT, 7% SDS, 40% glycerol and β-mercaptoethanol). The samples were boiled at 95 °C for 5 min, separated by SDS–polyacrylamide gel electrophoresis and transferred to the PVDF membranes (Merck Millipore). Membranes were blocked with 7% blocking reagent (Sigma-Aldrich) for 1 h and incubated with primary antibodies overnight at 4 °C. Surplus antibodies were washed with TBST (0.1% Tween 20) five times, and HRP-conjugated secondary antibodies were incubated for 1 h at room temperature. Blots were developed with Clarity Western ECL substrate (Bio-Rad). Antibodies are listed in Supplementary Table 1.

NFAT2 activity was quantified using ImageJ. The intensity of the dephosphorylated NFAT2 band (lower band; obtained after stimulation with thapsigargin (TG) (Calbiochem) in 1 µM for 10 min) was measured, and identical ROIs were applied to adjacent lanes. Data are expressed relative to dephosphorylated NFAT2 intensity.

### Fura-2 calcium imaging

The 10-mm circular cover glasses (MARIENFELD) were coated with 0.2% gelatin and poly-D-lysin (Sigma-aldrich) and incubated overnight at 37 °C. After washing with PBS, HUDEP-2 cells were allowed to attach for 15 min at 37 °C. In total, 1 µM of Fura-2, AM (Invitrogen) was loaded in cells at 37 °C for 30 min in Iscove’s modified Dulbecco’s medium (IMDM) medium. Cells were stimulated with 1 µM of TG or 10 U/ml of EPO. The Ca^2+^ imaging was performed at 340 nm and 380 nm in Tyrode solution (129 mM NaCl, 5 mM KCl, 1 mM MgCl_2_, 30 mM Glucose and 25 mM HEPES) containing either 0- or 2-mM Ca^2+^. Images were acquired using an IDX81 microscope (Olympus) equipped with a fluorescent lamp (Sutter Instrument Company), 40× objective lens (Olympus) and a charge-coupled device camera (HAMAMATSU). Data were analyzed with MetaMorph and Igor software.

### FACS analysis

Cells were fixed with 4% paraformaldehyde for 10 min. In total, 1 × 10^6 ^cells were stained with fluorescence-conjugated antibodies in 100 µl of FACS buffer (PBS with 3% FBS) for 30 min on ice. The stained samples were washed twice with FACS buffer and filtered through a 70-µm cell strainer (Falcon). Data were acquired on a FACS machine (BD Biosciences) and analyzed using FlowJo software. Antibodies used were anti-human CD71 and CD235a (BD Biosciences).

### Statistical analysis

Data are presented as mean ± s.e.m. The differences between two groups were evaluated using the unpaired two-tailed Student’s *t*-test. For comparisons involving more than two groups, one- or two-way analysis of variance (ANOVA) was used depending on the experimental design, followed by post hoc multiple comparison tests (for example, Tukey, Bonferroni, Dunnett). Statistical significance was defined at *P* < 0.05. Analyses were conducted using GraphPad Prism.

Please refer to the Supplementary Information for complete materials and methods.

## Results

### Orai1 is an erythroid Ca^2+^ channel that decreases as erythroblasts mature

**Fig. 1:**
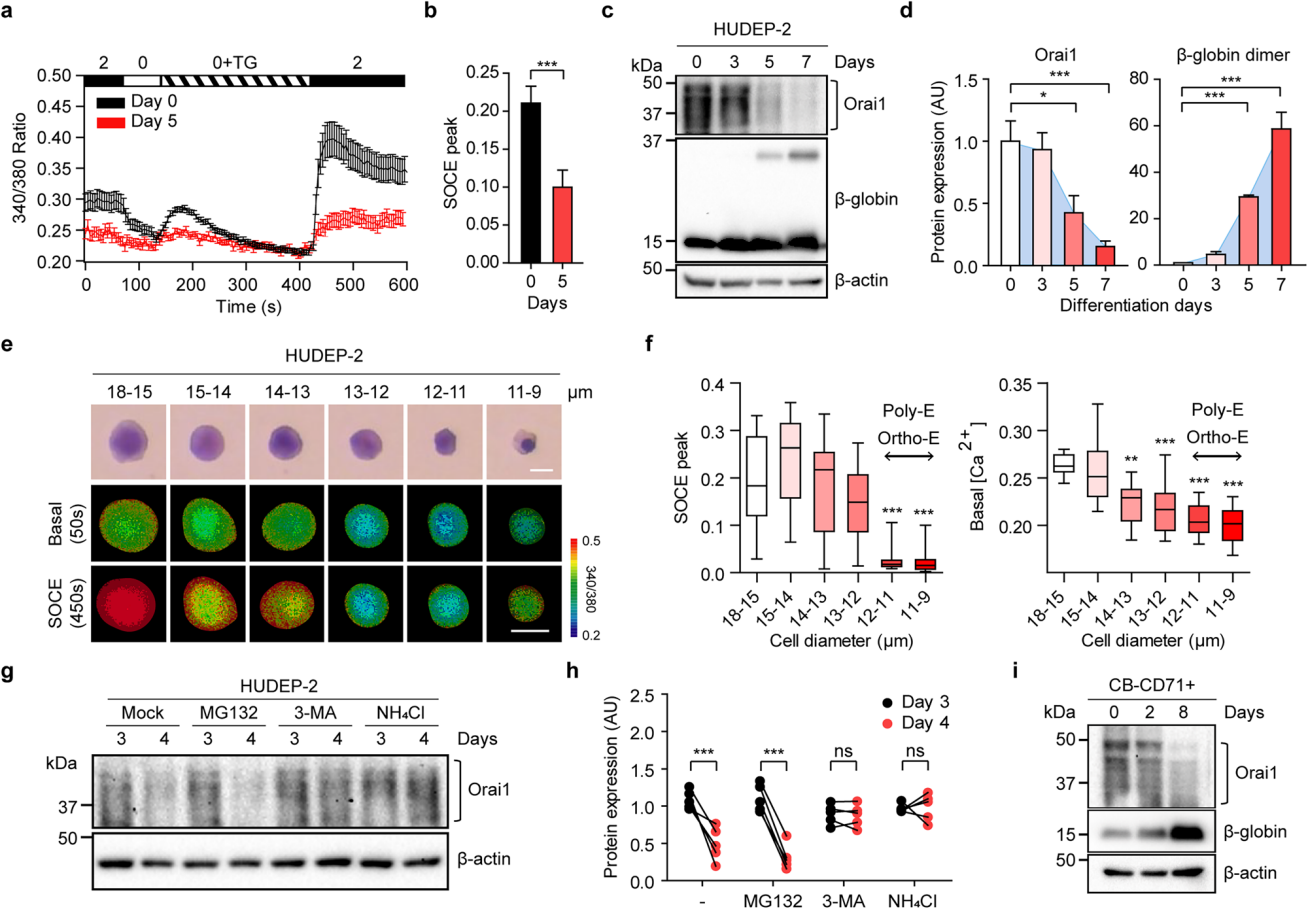
Orai1 Ca^2+^ channel decreases as erythroblasts mature. **a** Fura-2 Ca^2+^ imaging results of HUDEP-2 cells in maturation day 0 (black) and 5 (red); 1 µM of TG was treated after extracellular Ca^2+^ removal and 2 mM of Ca^2+^ solution re-added. **b** A comparison of the average SOCE peak level in **a** (*n* ≥ 50). **c** A representative blot image of Orai1, β-globin and β-actin expression during the erythropoiesis of HUDEP-2. **d** The densitometric quantification graph of Western blot bands (*n* = 5). **e** The May–Grunwald-stained (top) and fluorescent intensity ratio (340/380 nm) cell images (bottom) showing the cell size distribution of HUDEP-2 cells. The pixel intensity of the ratio image was quantitated and displayed at discrete levels. Pseudo-colors were indicated as a rainbow pattern (blue: lowest [Ca^2+^]i, 0.2; red: highest [Ca^2+^]i, 0.5). [Ca^2+^]i denotes intracellular Ca^2+^ concentration. The background outside the cell was masked in black for clarity; no image data within the cell were altered. Scale bar, 10 µm. **f** A box plot of SOCE peak (left) and basal [Ca^2+^]i (right) of a single cell distributed with the cell diameter (µm). **g** A western blot shows Orai1 under MG132, 3-MA and NH_4_Cl treatment. **h** The densitometric quantification graph of western blot bands (*n* = 4). **i** A representative blot image of Orai1, β-globin and β-actin expression during erythropoiesis of CB-derived CD71^+^ erythroblasts. Data are presented as mean ± s.e.m. Statistical analysis was performed using unpaired two-tailed *t*-test in **b**, one-way ANOVA with Dunnett’s post hoc test in **d** and **f** and two-way ANOVA with Bonferroni’s post hoc test in **h** (ns, *P* > 0.05; *P ≤ 0.05; **P ≤ 0.01; ***P ≤ 0.001).

To assess the impact of EPO signaling on terminal erythropoiesis, we induced the maturation of immortalized erythroblasts, HUDEP-2 cells^35^ under external EPO treatment, following a previously described method^36^ (Supplementary Fig. 1a). The efficient maturation was confirmed by the transition of CD71^+^ precursors to CD71^−^CD235a^+^ mature cells by day 7 (Supplementary Fig. 1b,c). Given the reported association between EPO and Ca^2+^ influx^37^, we first monitored SOCE during maturation with EPO treatment. At the immature stage (day 0), TG treatment in 0 mM Ca^2+^ solution, followed by the readdition of 2 mM Ca^2+^, caused a pronounced increase in intracellular Ca^2+^ levels, indicative of SOCE. This Ca^2+^ rise was suppressed by 2-APB, a SOCE antagonist (Supplementary Fig. 1d,e), confirming active SOCE in immature HUDEP-2 cells upon store depletion. Notably, after 5 days of maturation, SOCE and basal Ca^2+^ levels were significantly reduced in mature cells (Fig. 1a,b and Supplementary Fig. 1f).

As Orai1 predominantly mediates endogenous Ca^2+^ regulation via SOCE, we next monitored the Orai1 expression during maturation. Orai1 protein displayed multiple bands ranging from 33 to 50 kDa, as previously reported^38^, with its expression significantly reduced after 5 days of maturation (Fig. 1c,d). This reduction in Orai1 expression correlated with decreased SOCE activity (Fig. 1a,b), suggesting that Orai1 downregulation contributes to Ca^2+^ signaling during erythroblast maturation. Concurrently, β-globin dimer, a key marker of erythroid maturation, was markedly upregulated from day 5 (Fig. 1c,d). Consistently, *Orai1* transcript levels also decreased significantly as cells matured, whereas an increase in *Band3* expression further confirmed erythroid progression (Supplementary Fig. 1g). These findings indicate a reciprocal correlation between Orai1-mediated Ca^2+^ channel activity and terminal erythroblast maturation.

Subsequently, we analyzed Orai1-mediated SOCE across different stages of erythroblast maturation. As erythroblasts mature, they undergo size reduction, allowing HUDEP-2 cells to be classified into six diameter-based categories: 18–15, 15–14, 14–13, 13–12, 12–11 and 11–9 µm (Fig. 1e). This size ranges correspond to defined differentiation stages: proerythroblasts (18–15 µm), early basophilic erythroblasts (18–15 µm), late basophilic erythroblasts (15–12 µm), polychromatic erythroblasts (Poly-E; 15–11 µm) and orthochromatic erythroblasts (Ortho-E; 12–9 µm) (Supplementary Fig. 1h). After 5 days of maturation, the majority of cells measured less than 12 µm, indicating progression to the Poly-E and Ortho-E stages (Supplementary Fig. 1i). Notably, the SOCE activity considerably decreased in cells with diameters of 12–9 µm (Poly-E and Ortho-E stages), whereas basal Ca^2+^ level gradually declined as cell size decreased (Fig. 1f). These observations suggest that Orai1 downregulation is closely coupled with reduced SOCE activity and that this reduction may facilitate the precise progression of erythroid maturation by attenuating Ca^2+^-mediated signaling.

Previous studies have demonstrated that Orai1 is predominantly degraded through autophagic and lysosomal pathways rather than via the proteasome^39,40^. In addition, autophagy is known to be upregulated during erythroid differentiation^41^. On the basis of these findings, we posited that Orai1 may undergo degradation through the autophagy-lysosomal pathway during terminal erythropoiesis. To verify this, we treated HUDEP-2 cells with various proteolytic pathway inhibitors between three and four of maturation: a proteasome inhibitor (MG132), an autophagy inhibitor (3-methyladenine; 3-MA) and a lysosomal inhibitor (ammonium chloride; NH_4_Cl) (Fig. 1g). The cell viability following treatment was confirmed using an MTT assay (Supplementary Fig. 1j). Interestingly, the decrease in Orai1 levels was prevented by 3-MA and NH_4_Cl, but not by MG132 (Fig. 1g,h). These results indicate that Orai1 is preferentially degraded through the autophagic–lysosomal pathway during erythroblast maturation.

To extend our findings in a primary cell context, we investigated Orai1 expression in primary erythroblasts derived from UCB. CD71^+^ precursors were isolated and cultured using a defined maturation protocol (Supplementary Fig. 1k), enabling their development into mature CD71^−^CD235a^+^ cells over 8 days, as evidenced by increased β-globin expression (Fig. 1i and Supplementary Fig. 1l–n). Remarkably, on day 8, the matured cells exhibited a considerable reduction in Orai1 protein expression compared with those at days 0 and 2 (Fig. 1i and Supplementary Fig. 1n). Furthermore, *Orai1* mRNA levels were notably downregulated as the cells matured (Supplementary Fig. 1o). These results demonstrate that Orai1 expression substantially downregulated during terminal erythropoiesis in both immortalized and primary cells, suggesting that its reduction is a conserved feature of erythroblast maturation.

### EPO triggers Orai1-mediated Ca^2+^ entry in erythroblasts during early terminal erythropoiesis

**Fig. 2:**
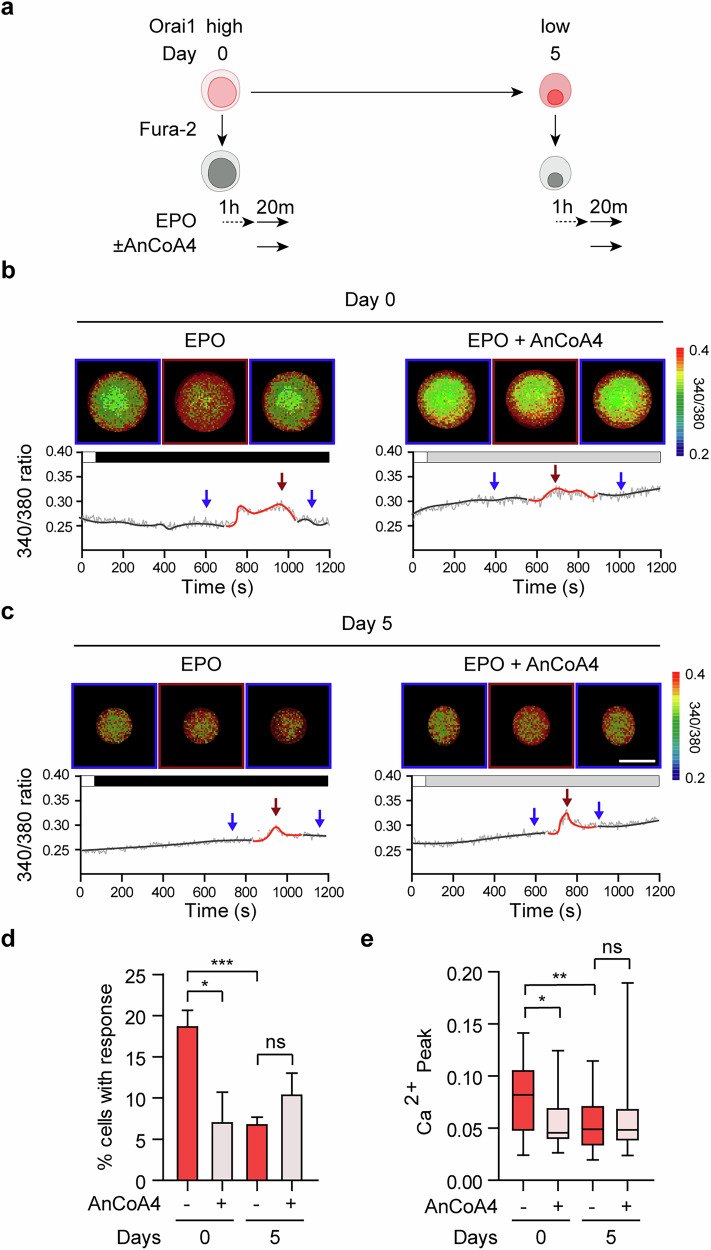
EPO triggers Orai1-mediated Ca^2+^ entry in early erythroblasts. **a** A schematic drawing of the experimental protocol for the EPO-mediated modification of Ca^2+^ influx. Cells were preincubated (1 h) in IMDM media without EPO. The cells were then exposed to EPO (50 U/ml) with or without AnCoA4 (10 µM). **b**, **c** Top: the fluorescent intensity ratio (340/380 nm) cell images of HUDEP-2 in EPO and AnCoA4 treated conditions in maturation day 0 (**b**) and day 5 (**c**). The pixel intensity of the ratio image was quantitated and displayed at discrete levels. Pseudo-colors were indicated as a rainbow pattern (blue: lowest [Ca^2+^]i, 0.2; red: highest [Ca^2+^]i, 0.4). [Ca^2+^]i denotes intracellular Ca^2+^ concentration. The background outside the cell was masked in black for clarity; no image data within the cell were altered. Bottom: a representative graph of calcium transients during the 1,200 s, under the indicated treatments. Scale bar, 10 µm. **d** The comparison of the average proportion of cells that had EPO-mediated Ca^2+^ influx shown in **b** and **c** (*n* ≥ 3). **e** The comparison of average Ca^2+^ peak shown in **b** and **c** (*n* ≥ 15). Data are presented as mean ± s.e.m. *P* values were calculated using an unpaired two-tailed *t*-test in **d** and **e**. (ns, *P* > 0.05; *P ≤ 0.05; **P ≤ 0.01; ***P ≤ 0.001).

In erythroid precursor cells, EPO induces Ca^2+^ influx by activating tyrosine kinase-dependent PLC-γ1^42^, which promotes ER Ca^2+^ release and subsequently triggers SOCE. To determine whether EPO induces Orai1-mediated SOCE during terminal erythropoiesis, we assessed EPO-triggered Ca^2+^ influx in HUDEP-2 cells treated with AnCoA4, a specific Orai1 inhibitor^43^. Fura-2 Ca^2+^ imaging was performed at two distinct stages of maturation: day 0, when Orai1 expression is high, and day 5, when Orai1 expression is low. To eliminate the residual EPO effects from the culture medium, cells were deprived of EPO for 1 h before stimulation with 50 U/ml of EPO for 20 min (Fig. 2a). EPO-induced Ca^2+^ responses differed markedly between the two phases, reflecting distinct maturation-dependent Ca^2+^ dynamics (Fig. 2b,c).

On day 0, approximately 18% of cells exhibited Ca^2+^ influx within 20 min of EPO stimulation, which decreased to less than 10% upon treatment with AnCoA4. Conversely, during day 5, the proportion of EPO-responsive cells was lower compared with day 0, and AnCoA4 had no significant effect on Ca^2+^ influx (Fig. 2d). Moreover, cells on day 0 displayed significantly greater Ca^2+^ transient amplitudes than those on day 5, and these transients were substantially impaired by Orai1 inhibition. Notably, AnCoA4 treatment had no discernible effect on Ca^2+^ entry during day 5 (Fig. 2e). These results indicate that, in the early stage, EPO activates Orai1-mediated Ca^2+^ entry in erythroblasts, whereas the reduction in Orai1 expression during the late stage leads to a diminished EPO-derived Ca^2+^ response. This underscores the role of Orai1 as a previously unrecognized mediator of EPO-driven Ca^2+^ dynamics in early erythroblast maturation.

### Inactivation of Orai1 enhances terminal erythropoiesis

**Fig. 3:**
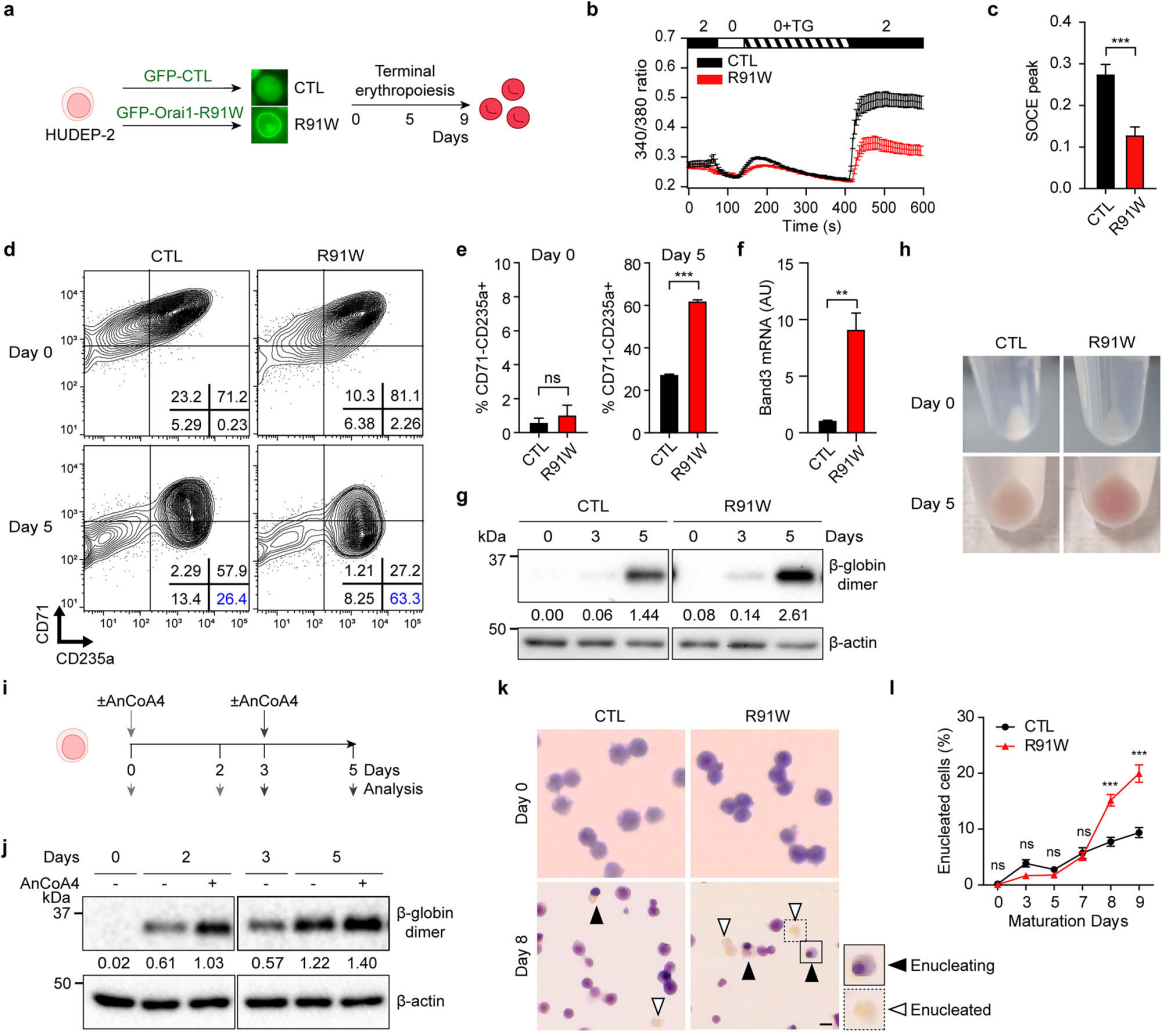
Inactivation of Orai1 enhances terminal erythropoiesis. **a** A schematic representation of the method for constructing N-terminally GFP-tagged Orai1 R91W and GFP control overexpressed HUDEP-2 cells. **b** The Fura-2 Ca^2+^ imaging results of GFP control (black) and Orai1 R91W (red) HUDEP-2 cells. **c** A comparison of the average SOCE peak level in **b** (*n* = 60). **d** A representative FACS analysis of anti-human CD71 and CD235a in Orai1 R91W overexpressed HUDEP-2 (right) and GFP control (left) on maturation day 0 (top) and 5 (bottom). **e** The proportion of CD71^−^CD235a^+^ in GFP control versus Orai1 R91W cells (*n* = 3). **f** mRNA expression levels of *Band3* in GFP control versus Orai1 R91W cells (*n* = 3). **g** The protein expression analysis of the dimer form of β-globin in GFP control and R91W mutant cells during terminal maturation. **h** Cell pellets of the GFP control (left) and GFP–Orai1–R91W (right) overexpressed HUDEP-2 cells in maturation day 0 (top) and 5 (bottom). **i** A schematic drawing of the experimental protocol for the AnCoA4 treatment and β-globin analysis. **j** The protein expression of a dimer form of β-globin under AnCoA4 treatment for 2 days from 0 and 3 days. **k** The May–Grunwald Giemsa staining of the GFP control and R91W mutant HUDEP-2. Enucleating cells are indicated with a black arrowhead, and enucleated cells are indicated with a white arrowhead. Scale bar, 10 µm. **l** Left: the proportion of enucleated cells depicted in **k**. Data are presented as mean ± s.e.m. *P* values were calculated using an unpaired two-tailed *t*-test in **c**, **e** and **f** and two-way ANOVA with Bonferroni posttests in **l** (ns, *P* > 0.05; **P ≤ 0.01; ***P ≤ 0.001).

To explore the functional role of Orai1 in erythroid maturation, we first generated stable HUDEP-2 cell lines expressing either a dominant-negative Orai1 mutant (GFP–Orai1–R91W)^44,45^ or GFP as a control via lentiviral transduction (Fig. 3a and Supplementary Fig. 2a). The R91W mutants exhibited a significant reduction in SOCE compared with control cells (Fig. 3b,c), confirming the effective inhibition of endogenous Orai1 activity. The stable expression of GFP–Orai1–R91W and GFP was further validated by immunoblotting throughout the 7-day maturation period (Supplementary Fig. 2b).

Erythroid maturation was evaluated by analyzing CD71 and CD235a expression before (day 0) and after 5 days of maturation. Surprisingly, the R91W mutants showed considerably enhanced maturation efficiency, with a higher proportion of CD71^−^CD235a^+^ matured cells at day 5 (CTL: ~26%, R91W: ~63%), whereas no significant difference was observed at day 0 (Fig. 3d,e). Furthermore, terminal erythroid markers^46^, including Band3, were significantly upregulated in R91W mutants (Fig. 3f and Supplementary Fig. 2c), suggesting that the inhibition of Orai1 activity promotes erythroid maturation.

Given that mice lacking SOCE retain hemoglobin levels even under leukemia-induced stress anemia^33^, we next examined whether Orai1 inhibition influences globin synthesis. The protein expression level of mature β-globin dimer was evaluated in R91W and control cells at different time points (days 0, 3 and 5) during a 5-day maturation period. R91W mutants exhibited enhanced globin maturation, with a higher expression of the β-globin dimer (Fig. 3g), especially on days 3 and 5, accompanied by more reddish cell pellets upon centrifugation compared with controls (Fig. 3h). To further confirm whether this increase was due to Orai1 inhibition, wild-type (WT) HUDEP-2 cells were treated with 10 µM AnCoA4 during two phases of maturation: days 0–2 and days 3–5 (Fig. 3i). The AnCoA4 treatment notably elevated β-globin dimer levels on day 2 and moderately increased them on day 5 (Fig. 3j). These findings indicate that Orai1 inhibition may accelerate β-globin synthesis and maturation, thereby promoting terminal erythroid maturation.

Enucleation, the final stage of erythroid maturation, was next examined to determine whether Orai1 inactivation would alter this critical process. The R91W mutants and control cells were subjected to erythroid maturation for 9 days, and the enucleation efficiency was assessed by evaluating the pool of enucleating and enucleated cells using Giemsa stain (Fig. 3k). R91W mutants displayed a higher proportion of enucleated cells beginning at day 8 compared with controls (Fig. 3l). Moreover, the enucleation was initiated earlier in the R91W mutants, as indicated by a greater number of enucleating cells at earlier time points (Supplementary Fig. 2d). These results suggest that Orai1 inactivation is sufficient to enhance the terminal maturation process, including β-globin synthesis and enucleation.

To further delineate the role of Orai1 activity, we investigated whether enforced channel activation would conversely impair erythroid maturation. HUDEP-2 cell lines stably overexpressing a constitutively active Orai1 mutant (Orai1-V102C) were established (Supplementary Fig. 3a–f), and terminal erythroid marker expression was evaluated at maturation day 3. Unlike the R91W mutants, Orai1-V102C cells exhibited a significantly reduced expression of these markers (Supplementary Fig. 3g), indicating that sustained Orai1 activation impairs the terminal differentiation. Overall, these findings represent the inaugural report demonstrating that the Orai1 as a critical determinant of terminal erythropoiesis, restraining differentiation and control the transition to mature RBCs.

### Orai1 decreases as hPS cell-derived CD71^+^ erythroblasts mature

**Fig. 4:**
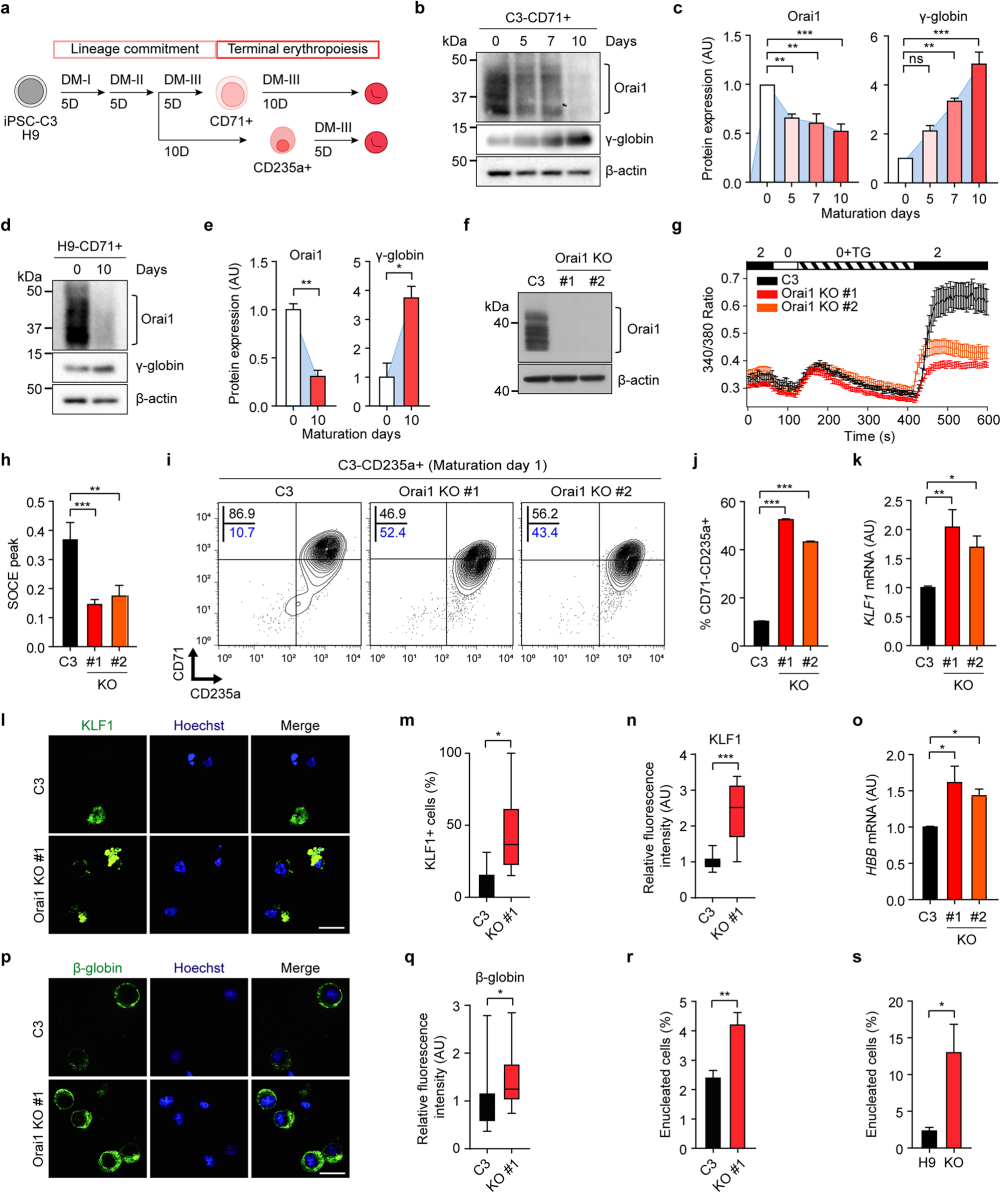
Orai1 inactivation enhances the maturation of hPS cell-derived erythroblasts. **a** A schematic representation of the maturation protocol of hPS cell-derived CD71^+^ and CD235a^+^ erythroblasts. **b**,**d** A representative blot image of Orai1, γ-globin and β-actin expression during maturation of C3-CD71^+^(**b**) and H9-CD71^+^(**d**) cells. **c**,**e** The densitometric quantification graph of the western blot bands, which are depicted in **b** (**c**) and **d** (**e**) (*n* = 3). **f** A representative western blot showing Orai1 protein in C3 and C3 Orai1 KOs. **g** The Fura-2 Ca^2+^ imaging results of C3 (black), Orai1 KO no. 1 (red) and KO no. 2 (orange) C3s. **h** A comparison of the average SOCE peak level (*n* ≥ 20). **i** A representative FACS analysis of CD71 and CD235a in C3- and Orai1-KO C3s-CD235a^+^ cells following maturation for 1 day. **j** The proportion of CD71^−^CD235a^+^ cells in C3 and Orai1-KO C3s derived erythroblasts shown in **i** (*n* = 3). **k** The relative mRNA expression level of *KLF1* of C3 and Orai1-KO cells on maturation day 1 (*n* = 3). **l** Immunofluorescent images showing KLF1 expression in C3 and Orai1-KO C3-CD235a^+^ cells. Scale bar, 20 µm. **m** The proportion of KLF1-positive cells in C3 versus Orai1-KO cells. **n** The relative fluorescence intensity of endogenous KLF1 in C3 versus Orai1-KO cells. **o** The relative mRNA expression level of *HBB* of C3 and Orai1-KO cells on maturation day 1 (*n* = 3). **p** Immunofluorescent images showing β-globin expression in C3 and Orai1-KO cells. Scale bar, 20 µm. **q** The relative fluorescence intensity of endogenous β-globin in C3 versus Orai1-KO cells (*n* = 25). **r**,**s** The proportion of enucleated cells in C3 (**r**) (*n* ≥ 31) and H9 (**s**) (*n* ≥ 13) versus Orai1-KO cells. Data are presented as mean ± s.e.m. *P* values were calculated using one-way ANOVA with Dunnett’s post hoc test in **c**, unpaired two-tailed *t*-test in **e**, **m**, **n**, **q**, **r** and **s** and one-way ANOVA with Tukey’s post hoc test in **h**, **j**, **k** and **o** (ns, *P* > 0.05; *P ≤ 0.05; **P ≤ 0.01; ***P ≤ 0.001).

hPS cells offer a promising source for generating transfusible RBCs. To extend our investigation of the novel role of Orai1 in terminal erythropoiesis to hPS cells, we established an erythroid differentiation protocol using two hPS cell lines: iPS cell derived from a group O/RhD^−^ donor (iPS cell-C3, hereafter referred to as C3)^47^, designated for the production of universal RBCs, and the human embryonic stem cell line H9. CD71^+^ erythroid precursors were isolated and matured into CD71^−^CD235a^+^ cells over 10 days using a defined protocol, following erythroid lineage commitment in sequential differentiation media I (DM-I), II (DM-II) and III (DM-III) (Fig. 4a and Supplementary Fig. 4a–c).

The subsequent analysis revealed that C3-derived CD71^+^ erythroblasts (C3-CD71^+^) exhibited substantial Orai1 protein expression at day 0, which markedly declined by days 5 and 10 of maturation (Fig. 4b,c). Similarly, there was a dramatic reduction in Orai1 protein levels in H9-derived CD71^+^ erythroblasts (H9-CD71^+^) after 10 days of maturation (Fig. 4d,e). These findings indicate that Orai1 undergoes a consistent decrease during the terminal maturation of hPS cell-derived erythroblasts, mirroring the pattern observed in HUDEP-2 and UCB-derived erythroblasts. This consistent downregulation prompted us to further examine whether Orai1 inactivation facilitates terminal erythropoiesis in these cells, akin to its role in immortalized HUDEP-2 cells.

### Ablation of Orai1 enhances the maturation of hPS cell-derived erythroblasts

To assess whether Orai1 inactivation improves the maturation of hPS cell-derived erythroblasts, we generated Orai1-KO hPS cells using a homology-directed repair-based Cas9 system. Orai1-KO hPS cell lines were established by inserting an mCherry transgene into Exon 1 of the Orai1 locus (Supplementary Fig. 5a). Successful KO was confirmed by immunoblotting (Fig. 4f and Supplementary Fig. 8a) and PCR (Supplementary Fig. 5b,c). Orai1-KO cells exhibited significantly reduced SOCE compared with WT cells (Fig. 4g,h and Supplementary Fig. 8b,c). Importantly, Orai1 depletion did not compromise pluripotency markers (Tra-1-60, SSEA4, OCT4, NANOG and SOX2) or three germ layer markers (mesoderm: SMA and MIXL; ectoderm: nestin; endoderm: Sox17), ensuring that Orai1-KO did not affect essential features of hPS cells (Supplementary Fig. 6a–c). In addition, no off-target effects were observed (Supplementary Fig. 5d).

To evaluate terminal maturation, CD235a⁺ erythroid cells—representing late-stage populations—were isolated from Orai1-KO and WT cells, allowing us to examine the effect of Orai1 loss on erythroid maturation (Fig. 4a and Supplementary Fig. 7a). One day after isolation, Orai1-KO cells displayed enhanced maturation efficiency compared with WT cells, showing a higher population of CD71^−^CD235a^+^ mature cells (Fig. 4i,j and Supplementary Fig. 7b). This enhanced maturation of Orai1-KO hPS cells was further intensified at 5 days post isolation (Supplementary Figs. 7c–e and 8d,e).

KLF1, a master transcription factor of terminal erythropoiesis, is critical for augmenting the maturation and stability of hPS cell-derived RBCs^13^. Notably, Orai1-KO CD235a^+^ cells showed increased KLF1 expression at both mRNA (Fig. 4k) and protein levels compared with WT cells (Fig. 4l–n and Supplementary Fig. 8f–h). In particular, Orai1-KO cells exhibited a higher proportion of KLF1-positive cells with elevated KLF1 expression at the single-cell level compared with WT cells. These results imply that Orai1 inactivation induces KLF1 expression, consequently promoting the terminal maturation of hPS cell-derived erythroblasts.

The production of functional RBCs from hPS cells remains challenging owing to inefficient maturation, including low levels of adult β-globin expression and limited enucleation. To investigate whether Orai1-KO hPS cells could overcome these limitations, we assessed β-globin expression in CD235a^+^ cells. Orai1-KO cells exhibited higher β-globin mRNA levels (Fig. 4o). Immunocytochemistry further confirmed enhanced β-globin expression, with a greater proportion of Orai1-KO cells exhibiting strong staining, whereas most WT cells showed only weak signals (Fig. 4p,q and Supplementary Fig. 8i,j), indicating a qualitative enhancement in globin production. Furthermore, enucleation efficiency, another hallmark of terminal erythropoiesis, was increased in Orai1-KO CD235a^+^ cells, as demonstrated by May–Grunwald Giemsa staining (Fig. 4r,s and Supplementary Fig. 8k). Together, these data underscore the pivotal role of Orai1 inactivation in promoting the generation of functional RBCs from hPS cell-derived erythroblasts.

### NFAT2 inhibits the transcription of KLF1

**Fig. 5:**
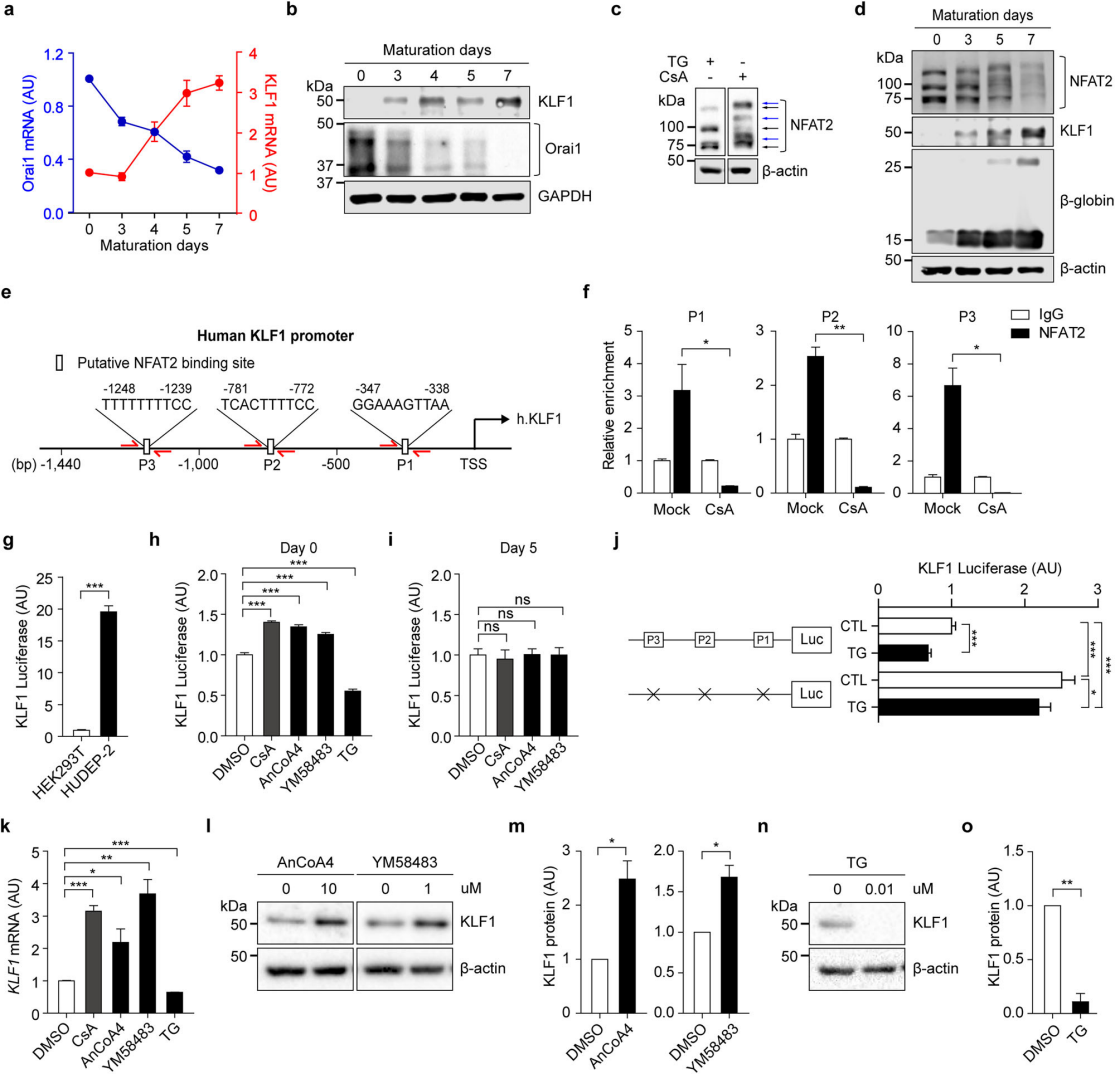
Orai1-mediated NFAT2 inhibits KLF1 transcription. **a** The RT–qPCR results of KLF1 and Orai1 during maturation of HUDEP-2 (*n* = 3). **b** A western blot analysis of KLF1, Orai1 and β-actin for maturation day 7. **c** A western blot analysis of NFAT2 and β-actin in HUDEP-2 under 1 µM TG and 1 µM CsA treatment. **d** A western blot analysis of NFAT2, KLF1 and β-globin during maturation. **e** Three putative NFAT2 binding sites in the KLF1 promoter. The half arrow indicates the primer binding sites for P1, P2 and P3. **f** The ChIP analysis determines NFAT2 binding to the KLF1 promoter with or without CsA (0.1 µM, 24 h) (*n* = 3). **g** A comparison of KLF1 luciferase in HEK293T and HUDEP-2 cells (*n* ≥ 7). **h**,**i** KLF1 luciferase assay under CsA (0.1 µM), AnCoA4 (10 µM), YM58483 (1 µM) and TG (0.01 µM) treatment at maturation day 0 (**h**) and 5 (**i)** (*n* = 3). **j** The KLF1 luciferase assay of NFAT2 binding site mutants and WT under TG (0.1 µM) treatment. **k** The mRNA expression level of *KLF1* under CsA (0.1 µM), AnCoA4 (10 µM), YM58483 (1 µM) and TG (0.01 µM) treatment at maturation day 0 (*n* = 3). **l** A western blot showing an increase of endogenous KLF1 protein in cells treated with AnCoA4 (10 µM) and YM58483 (1 µM) for 2 days. **n** A western blot showing a decrease of endogenous KLF1 protein in cells treated with TG (0.01 µM) for 24 h. **m**,**o** The densitometric quantification graph of western blot bands in **l** (**m**) and **n** (**o**) (*n* = 3). Data are presented as mean ± s.e.m. *P* values were calculated using an unpaired two-tailed *t*-test (ns, *P* > 0.05; *P ≤ 0.05; **P ≤ 0.01; ***P ≤ 0.001).

We next explored the molecular mechanisms connecting the Orai1 inactivation to enhanced terminal maturation. A recent study reported that Orai1 regulates the expression of KLF families^48^. In addition, elevated intracellular Ca²⁺ levels have been shown to suppress *KLF1* expression and its downstream targets. Consistent with these, our results demonstrated that CD235a^+^ cells derived from Orai1-KO hPS cells exhibited significantly increased KLF1 expression (Fig. 4k–n). Conversely, HUDEP-2 cells overexpressing the constitutively active Orai1-V102C mutant showed markedly reduced *KLF1* mRNA and protein levels (Supplementary Fig. 9a,b). To further investigate this relationship, we assessed the temporal expression patterns of KLF1 and Orai1 in HUDEP-2 cells throughout erythroid maturation (days 0, 3, 4, 5 and 7). KLF1 exhibited an inverse expression pattern to Orai1 at both transcript and protein levels, remaining low during days 0–3 and increasing substantially during days 5–7 (Fig. 5a,b and Supplementary Fig. 10a). These findings suggest that Orai1 negatively regulates KLF1 expression during terminal erythropoiesis.

NFAT2, a Ca^2+^-dependent transcription factor regulated by Orai1^49^, has been implicated in the regulation of KLF1 transcription in murine erythroblasts^24^. To explore the role of NFAT2 in the Orai1-mediated inhibition of KLF1 during terminal erythropoiesis, we first characterized the intrinsic activation profile of NFAT2 in erythroid progenitors. NFAT2 activity was examined in HUDEP-2 cells under pharmacological modulation using western blot analysis (Fig. 5c) with an antibody validated for NFAT2 specificity (Supplementary Fig. 10b). Treatment with TG induced the appearance of three distinct dephosphorylated NFAT2 isoforms^50,51^, indicative of its activation. By contrast, exposure to cyclosporin A (CsA), a blocker of Ca^2+^-calcineurin-dependent NFAT2 activation, resulted in the accumulation of phosphorylated (inactive) NFAT2, confirming that NFAT2 activity in erythroid progenitors is dynamically regulated by intracellular Ca^2+^ signaling.

To investigate the temporal dynamics of NFAT2 activity during terminal erythropoiesis, we analyzed its activation status in HUDEP-2 cells at multiple time points (days 0, 3, 5 and 7) (Fig. 5d). At day 0, before differentiation, three distinct dephosphorylated NFAT2 isoforms were detected, as observed under TG treatment (Fig. 5c). As differentiation progressed, a gradual increase in phosphorylated (inactive) NFAT2 was observed, reflected by an upward shift of the NFAT2 band on western blot. Quantitative analysis, based on the ratio of dephosphorylated to phosphorylated forms, revealed a progressive decline in NFAT2 activity during late stages of maturation (Supplementary Fig. 10c). This decrease paralleled the downregulation of Orai1, suggesting that Orai1 may modulate NFAT2 activity through Ca^2+^-dependent signaling. Notably, this reduction in NFAT2 activity was accompanied by a marked increase in KLF1 protein levels, supporting the role of NFAT2 as a negative regulator of KLF1 expression during terminal erythropoiesis.

To test whether Orai1 activity is necessary for NFAT2-mediated repression of KLF1, we analyzed HUDEP-2 cells expressing the Orai1 R91W mutant alongside WT controls. In WT cells, TG stimulation activated NFAT2, as shown by the shift to dephosphorylated isoforms on western blot. By contrast, R91W mutants exhibited minimal response to TG, with NFAT2 largely remaining in its phosphorylated state. The basal NFAT2 activity was also significantly lower in R91W mutants (Supplementary Fig. 10d,e), demonstrating that Orai1 is essential for NFAT2 activation in erythroblasts. Correspondingly, R91W mutants displayed elevated *KLF1* mRNA levels compared with WT cells (Supplementary Fig. 10f), highlighting a negative role of Orai1-activated NFAT2 signaling on KLF1 expression.

To determine whether NFAT2 directly regulates KLF1 transcription by binding to its promoter, we identified three putative NFAT2 binding sites (P1, P2, P3) within ~1,440 bp upstream of the KLF1 transcription start site using PROMO 3.0.2 software (Fig. 5e and Supplementary Fig. 10g). Chromatin immunoprecipitation (ChIP) assays revealed that endogenous NFAT2 binds to all three predicted sites with higher affinity than the IgG control. Notably, this binding was reduced upon NFAT2 inactivation by CsA, confirming a direct NFAT2 interaction with the KLF1 promoter region (Fig. 5f).

Building on the ChIP results, we next assessed the transcriptional effects of NFAT2 binding using a luciferase reporter containing the WT KLF1 promoter with all three identified NFAT2 binding sites. Robust luciferase activity was observed in HUDEP-2 cells but not in HEK293T cells, confirming erythroid-specific activation of the KLF1 promoter (Fig. 5g). Pharmacological modulation of NFAT2 showed that inhibition with CsA and Orai1 inhibitors (AnCoA4 and YM58483) enhanced promoter activity at day 0, whereas activation by TG suppressed luciferase activity (Fig. 5h). These results suggest that Orai1–NFAT2 signaling represses KLF1 promoter activation during the early phase of terminal erythropoiesis. This inhibitory effect, however, diminished at the later maturation phase (Fig. 5i), probably owing to the reduced involvement of Orai1 in KLF1 transcription during advanced erythropoiesis. To further validate the functional contribution of NFAT2 binding sites to this repression, we constructed a mutant reporter in which all three NFAT2 motifs were disrupted. Under basal conditions, this mutant exhibited significantly higher luciferase activity than the WT construct, indicating that the loss of NFAT2 binding relieves transcriptional repression. Upon TG stimulation, the mutant promoter exhibited only a modest decrease in activity (Fig. 5j), demonstrating that NFAT2-mediated suppression largely depends on its direct binding to these motifs.

Finally, we investigated whether the inhibitory effect of Orai1–NFAT2 signaling on KLF1 promoter activation extends to KLF1 mRNA and protein expression. The treatment of HUDEP-2 cells with CsA or Orai1 inhibitors increased KLF1 mRNA and protein levels (Fig. 5k–m), whereas TG treatment substantially reduced both transcript and protein levels (Fig. 5k,n,o). These findings demonstrate that attenuation of Orai1–NFAT2 signaling is sufficient to induce KLF1 expression at the transcriptional and translational levels. Collectively, these results establish Orai1-mediated NFAT2 signaling as a novel negative regulator of KLF1 expression. The spontaneous downregulation of Orai1–NFAT2 activity during terminal erythropoiesis facilitates the induction of KLF1 expression, thereby driving the generation of mature RBCs. These findings highlight the Ca^2+^-mediated transcription factor, NFAT2 as a key modulator of KLF1 expression during erythropoiesis.

### EPO orchestrates dual mechanisms of KLF1 transcription, encompassing Orai1–NFAT2 downregulation followed by STAT5 maintenance

**Fig. 6:**
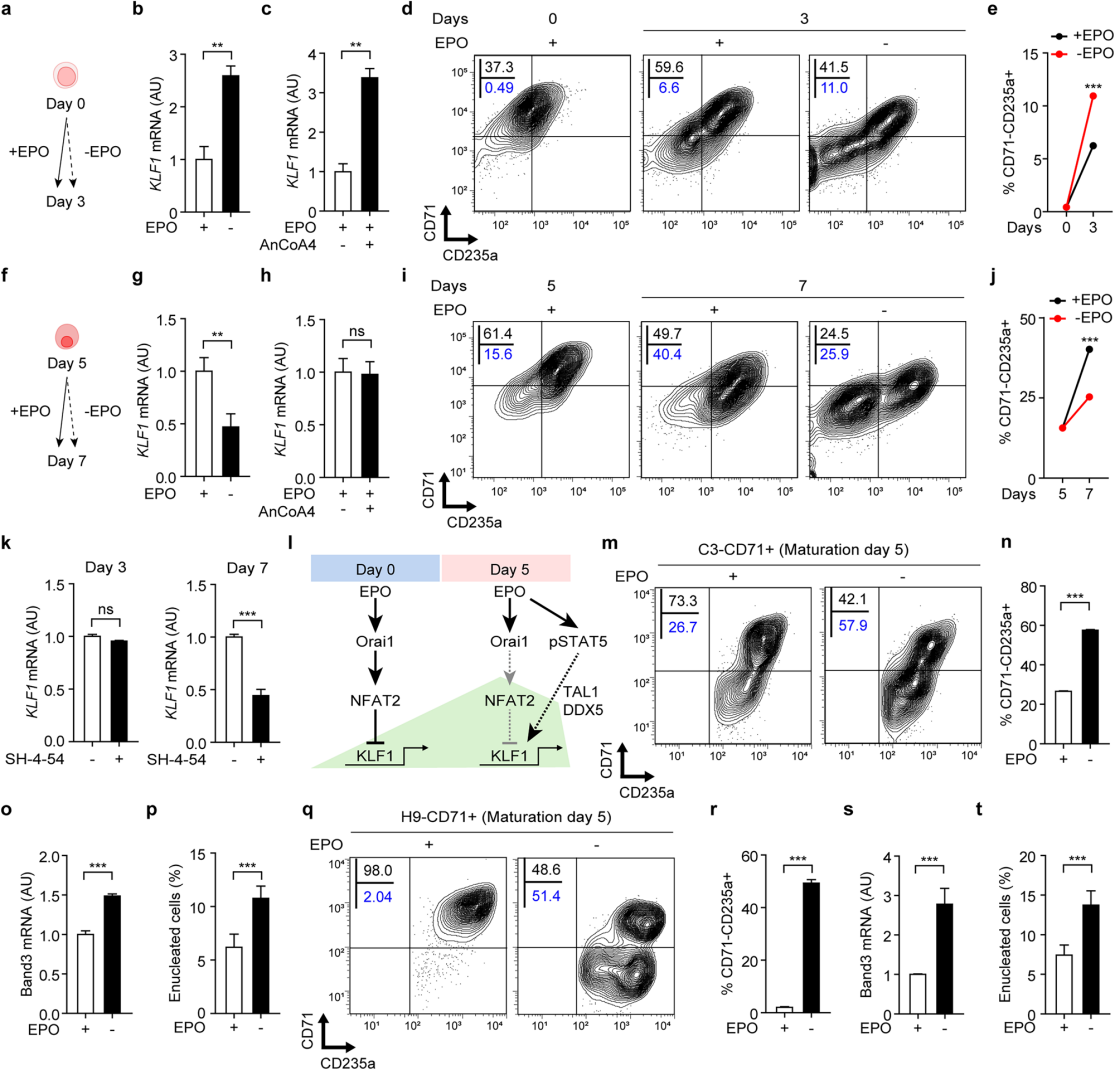
EPO-Orai1-KLF1 pathway shifts toward EPO–STAT5–KLF1 pathway, potentiating maturation. **a**,**f** A schematic drawing of the experimental protocol for EPO deprivation during the day 0 (**a**) and day 5 (**f**) phases of HUDEP-2. **b**,**c** The relative level of *KLF1* mRNA of HUDEP-2 in normal versus EPO-eliminated (**b**) and AnCoA4 (10 µM) (**c**)-treated conditions from day 0 to 3 (*n* = 3). **d**,**i** The representative FACS analysis of CD71 and CD235a of HUDEP-2 in EPO-deprived or normal conditions during days 0–3 (**d**) and days 5–7 (**i**). **e**,**j** The proportion of CD71^−^CD235a^+^ cells in normal versus EPO-eliminated conditions depicted in **d** (**e**) and **i** (**j**). **g**,**h** The relative level of *KLF1* mRNA of HUDEP-2 in normal versus EPO-eliminated (**g**) and AnCoA4 (10 µM) (**h**)-treated conditions from day 5 to 7 (*n* = 3). **k** The RT–qPCR result of KLF1 during differentiation with or without SH-4-54 in differentiation day 3 and day 7 (*n* = 3). **l** A schematic drawing of a model of the potential role for Orai1 and STAT5 in KLF1 transcription during terminal erythropoiesis. **m**,**q** A representative FACS analysis of CD71 and CD235a of C3-CD71^+^ (**m**) and H9-CD71^+^ (**q**) cells in EPO-deprived or normal conditions following maturation for 5 days. **n**,**r** The proportion of CD71^−^CD235a^+^ cells in normal versus EPO-eliminated conditions (*n* = 3). **o**,**s** The RT–qPCR result of Band3 of C3-CD71^+^ (**o**) and H9-CD71^+^ (**s**) cells in the normal versus EPO-eliminated condition (*n* = 3). **p**,**t** The proportion of enucleated cells of C3-CD71^+^ (**p**) and H9-CD71^+^ (**t**) cells in normal versus EPO-eliminated condition (*n* = 3). Data are presented as mean ± s.e.m. *P* values were calculated using an unpaired two-tailed *t*-test (ns, *P* > 0.05; *P ≤ 0.05; **P ≤ 0.01; ***P ≤ 0.001).

Our findings thus far demonstrate that EPO activates the Orai1 channel, with Orai1-dependent NFAT2 inhibiting KLF1 expression and decelerating erythroid maturation. To further elucidate the role of EPO in KLF1 transcription in an Orai1-dependent manner, we removed EPO during days 0–3 of HUDEP-2 cells and assessed *KLF1* mRNA levels (Fig. 6a). Interestingly, EPO removal increased *KLF1* mRNA levels (Fig. 6b), accompanied by reduced NFAT2 nuclear translocation (Supplementary Fig. 11a), confirming the inactivation of the Orai1–NFAT2 pathway. Similarly, treatment with AnCoA4 in the presence of EPO also elevated *KLF1* mRNA levels (Fig. 6c). Moreover, EPO removal during this phase enhanced maturation, yielding a higher proportion of CD71^−^CD235a^+^ cells (Fig. 6d,e). These findings suggest that EPO-mediated activation of the Orai1–NFAT2 pathway downregulates KLF1 expression and may play a role in limiting erythroid maturation when Orai1 expression is high.

To extend our findings, we examined the role of EPO in KLF1 transcription during days 5–7, when Orai1 activity decreases. EPO was removed, and *KLF1* mRNA levels were assessed in HUDEP-2 cells during this phase (Fig. 6f). Unlike the days 0–3, the absence of EPO significantly reduced *KLF1* mRNA levels (Fig. 6g), whereas AnCoA4 treatment had no effect (Fig. 6h). Moreover, cells matured without EPO exhibited reduced maturation efficiency compared with EPO-treated controls (Fig. 6i,j). These results suggest that when Orai1 expression is low, KLF1 transcription is sustained by an alternative, Orai1-independent EPO signaling pathway that supports terminal erythroid maturation.

To further elucidate the downstream effectors responsible for maintaining KLF1 transcription during the late stage, we investigated whether EPO regulates known positive regulators of KLF1, including TAL1 and DDX5. HUDEP-2 cells were cultured from day 5 to day 7 of erythroid differentiation with or without EPO supplementation, and mRNA levels of *TAL1* and *DDX5* were assessed (Supplementary Fig. 11b,c). Notably, the absence of EPO during this stage resulted in a marked reduction in both TAL1 and DDX5 expression, suggesting that these transcriptional regulators may contribute to Orai1-independent, EPO-dependent maintenance of KLF1 expression during late-stage erythroid maturation. Supporting this, TAL1 expression progressively increased over time, whereas DDX5 peaked at day 5 (Supplementary Fig. 11d,e), indicating their enhanced involvement during the late stage.

Given that STAT5 is a well-established downstream effector of EPO signaling and has been reported to positively regulate DDX5 expression^19^, we investigated whether STAT5 contributes to KLF1 expression as part of the Orai1-independent EPO signaling pathway. HUDEP-2 cells were treated with SH-4-54, a STAT5 inhibitor^52^, during both days 0–3 and days 5–7. STAT5 inactivation significantly reduced *KLF1* mRNA levels during days 5–7 but had no effect during days 0–3 (Fig. 6k). SH-4-54 treatment led to a marked decrease in STAT5 phosphorylation (Supplementary Fig. 11f), with a similar reduction observed upon EPO removal (Supplementary Fig. 11g). In addition, STAT5-inhibited cells exhibited a reduced maturation efficiency, as indicated by a slightly lower proportion of CD71^−^CD235a^+^ cells compared with controls (Supplementary Fig. 11h,i). These results indicate that an EPO-activated STAT5 signaling pathway is required to maintain *KLF1* mRNA expression during the late stage, thereby supporting the final stages of erythroid maturation. Collectively, these data propose a dual regulatory mechanism for EPO-mediated modulation of KLF1 transcription: an initial downregulation mediated by Orai1–NFAT2, followed by sustained expression via a STAT5-dependent pathway during advanced maturation (Fig. 6l).

Finally, we investigated whether eliminating EPO during early stage expedites terminal maturation of hPS cell-derived CD71^+^ erythroblasts by reducing Orai1 activity. CD71^+^ cells from C3 and H9 hPS cell lines were cultured with or without EPO from day 0 to 5, and maturation efficiency was evaluated. Consistent with findings in HUDEP-2 cells, EPO removal enhanced cell maturation, as evidenced by a higher proportion of CD71^−^CD235a^+^ cells (Fig. 6m,n,q,r) and increased *Band3* mRNA levels (Fig. 6o,s). Furthermore, cells that matured without EPO exhibited significant progression in enucleation compared with EPO-treated controls (Fig. 6p,t and Supplementary Fig. 11j,k). These results confirm that the inhibitory role of EPO signaling on maturation during the early stage extends to hPS cells beyond immortalized cell lines. In addition, EPO elimination may serve as a potential strategy to increase mature RBC production from hPS cell-derived CD71^+^-erythroid cells.

## Discussion

Considerable progress has been achieved in elucidating molecular mechanisms and signaling pathways essential for RBC maturation. This enhances our understanding and highlights the need for further advancements to enable in vitro RBC production for transfusion. Our study contributes to this progress by revealing Orai1 as a novel Ca^2+^ channel in controlling erythroid maturation, translating EPO signals into mechanistic decisions for either erythroblast maintenance or maturation. Quantitative changes in Orai1 act as a regulatory toggle, translating EPO signals into distinct outcomes: in its ‘on’ state, Orai1 expression leads to NFAT-mediated suppression of KLF1, maintaining erythroblast identity. Conversely, in its ‘off’ state, reduced Orai1 expression lifts this inhibitory effect, redirecting EPO signaling to sustain KLF1 expression and drive terminal erythropoiesis.

Orai1, along with Orai2 and Orai3, constitutes the Orai channel family, which regulates calcium signaling in various cellular processes. The incomplete attenuation of SOCE observed with the forced inactivation of Orai1 using R91W mutants or complete KO (Figs. 3b,c and 4g,h) suggests that Orai2 or Orai3 may partially compensate for the loss of Orai1 in CD71^+^ erythroblasts. Moreover, the modest increase in TG-mediated SOCE and EPO-induced calcium influx during late terminal maturation of HUDEP-2 (Figs. 1a,b and 2c–e) implies a potential role for Orai2 or Orai3 in regulating late-stage terminal erythropoiesis. Calcium signaling is critical regulatory pathway in late-stage erythropoiesis, particularly in processes such as erythrocyte enucleation, which depend on Ca^2+^–calmodulin signaling through the calmodulin–myosin II pathway^53^. Moreover, during reticulocyte maturation, nonessential proteins are sequestered within internal vesicles of multivesicular bodies and released as exosomes into the extracellular milieu^54^. Savina et al. demonstrated that calcium-dependent mechanisms regulate multivesicular body biogenesis and exosome release are in human K562 cells^55^. However, the specific roles of Orai2 or Orai3 in these intricate processes remain incompletely understood. Although our study highlights the importance of Orai1 in erythroid maturation, further investigation is warranted to elucidate the precise contributions of Orai2 or Orai3 to terminal erythropoiesis.

The production of functional RBCs in vitro provides a safe and reliable alternative to donor blood and serves as carriers of therapeutic molecules. Advances in culture protocols have enabled the derivation of RBCs from CD34^+^ hematopoietic stem and progenitor cells, hPS cells and immortalized erythroblasts^56^. However, current protocols face inefficiencies in the final stages of erythroid development, including β-globin expression and enucleation^57^. Moreover, the embryonic character remains a major issue within hPS cell. Studies have explored inhibition of the Hedgehog (Hh) signaling in lineage specification to shift the embryonic status to an adult stage^58,59^. Moreover, research has focused on transcriptional manipulation, such as overexpressing of BCL11A-XL and/or KLF1 to enhance β-globin expression and leveraging KLF1-overexpressed macrophages as erythroblastic islands to promote enucleation^60^. Despite progress, challenges remain in establishing efficient pipelines for adult-type RBC production. Our findings on Orai1–Ca^2+^ signaling provide novel insights, demonstrating its potential to enhance the production of functional erythrocytes from hPS cells, contributing to greater efficacy in cell replacement therapies.

The main limitations of this study are as follows: although Orai1 inactivation enhanced enucleation and β-globin expression, these cells still fell short of complete terminal maturation observed in fully mature erythrocytes. The potential impact of Orai1 loss on proliferative capacity during erythroid expansion also remains to be determined. Furthermore, because our findings were obtained exclusively in vitro, in vivo studies are needed to validate whether Orai1 modulation exerts comparable effects within the hematopoietic niche. Finally, given the critical role of Orai1 in T, B and NK cell function, lineage-specific approaches using terminally committed erythroid precursors (for example, CD71⁺ erythroblasts) will be important to minimize immunological side effects.

## Supplementary information

Supplementary Information
