# Supplementary material for: Orai1 acts as a novel Ca2+ signal switch, balancing erythropoiesis through KLF1 regulation
Source: Exp Mol Med. 2026 Mar 4;58(3):696–708. doi: 10.1038/s12276-026-01651-0 (PMC13049018; doi:10.1038/s12276-026-01651-0)
Supplement: Supplementary file 1 — Supplementary Information [file 12276_2026_1651_MOESM1_ESM.pdf]

Supplementary Information for

Orai1 acts as a novel Ca^{2+} signal switch, balancing erythropoiesis through KLF1 regulation

This file includes:

Supplementary materials and methods

Supplementary Table 1 to 2

Supplementary Figures 1 to 11

Supplementary materials and methods

H9 cell

The human embryonic stem cells (H9 line) used in this study were purchased from WiCell, a globally recognized provider of stem cell resources. The cells were acquired under proper authorization and were used exclusively for research purposes in accordance with institutional and ethical guidelines.

HUDEP-2 Cell culture

HUDEP-2 cell expansion medium consisted of StemSpan SFEM (STEMCELL Technologies) supplemented with 1 μg/ml doxycycline (Clontech), 1 μM dexamethasone (Sigma-Aldrich), 5 U/ml EPO (PeproTech), and 50 ng/ml SCF (PeproTech). For erythroid differentiation, cells cultured in IMDM (Sigma-Aldrich) medium containing 5 % human AB serum (Sigma-Aldrich), 400 μg/ml holo-transferrin (Sigma-Aldrich), 10 μg/ml human insulin (Sigma-Aldrich), 5 U/ml EPO, 50 ng/ml SCF, 1 μg/ml doxycycline, 3 U/ml heparin (Sigma-Aldrich), and 1 % L-glutamine (Gibco) for 5 days. For further maturation, cells were cultured in a doxycycline-removed medium for 2 to 3 more days.

RBC differentiation from cord blood

CB cells were cultured with IMDM (Sigma-Aldrich) supplemented with 15% FBS (EMD Millipore), 2 mM L-glutamine, 1 mM sodium pyruvate, 1x BIT, 25 ng/ml IL-3 (PeproTech), 100 ng/ml SCF, 100 ng/ml Flt3-L (PeproTech), and 100 ng/ml TPO (PeproTech). $CD34^+$-UCB cells sorted with the Microbeads kit were differentiated into RBC using IMDM medium supplemented for the first 6 to 8 days with 5 % h.AB serum, 3 U/ml heparin, 10 μg/ml insulin, 60 ng/ml SCF, 5 ng/ml IL3, 3 U/ml EPO, 1 μM Hydrocortisone, 200 μg/ml holo-transferrin. After 6 to 8 days, the differentiation medium was changed to IMDM medium supplemented with 5% h.AB serum, 3 U/ml heparin, 10 μg/ml insulin, 60 ng/ml SCF, 3 U/ml EPO, 300 μg/ml holo-transferrin.

hPSCs characterization

Fluorescence-activated cell sorting (FACS)

Cells were dissociated with enzyme-free Cell Dissociation Buffer (Gibco) and stained with conjugated antibodies. For transcription factors, a Fixation and Permeabilization kit (BD Biosciences) was used before antibody staining. Labeled cells were analyzed by flow cytometry on a BD FACSAriaTM II (BD Biosciences) and then using FlowJo software (BD Biosciences). The antibodies used for flow cytometry: Oct3/4 (BD Biosciences), Nanog (BD Biosciences), Sox2 (BD Biosciences), SSEA-4 (BD Biosciences), Tra-1-60 (BD Biosciences).

Immunocytochemistry (ICC)

Cells were fixed using Fixation Buffer (BD Biosciences) and stained with previously used antibodies. If permeabilization was required, cells were incubated with a Fixation and Permeabilization kit (BD Biosciences). The cells were stained with conjugated antibodies diluted in BD perm/wash buffer and incubated overnight at 4 °C. Nuclei were counterstained with Hoechst stain solution. Images were acquired on fluorescence microscopy (Leica).

Characterization of Orai1$^{-/-}$ knockout human iPSC lines

Genotyping of the Orai1$^{-/-}$ knockout cell was performed using a forward primer of the exterior 5' arm (5'- caggaaccggtcacctcaatacgtatcagatg -3') and a reverse primer of the exterior 3' arm (5'- gagcgagactccgtcttggaac -3') in Orai1 gene. Western blotting proceeded on standard protocols. Anti-Orai1 antibody (Santa Cruz Biotechnology) or anti-Actin (Santa Cruz Biotechnology).

MACS cell separation

The CD71$^+$ or CD235a$^+$ blood cells were sorted using a MACS cell separation (Miltenyi Biotec). At day 15, suspended blood cells were collected using a cell strainer and resuspended with MACS buffer. Cells were reacted with 20μL of anti-CD71 (Miltenyi Biotec) or CD235a microbead (Miltenyi Biotec) per 10^7 cells for 15 minutes at 4 °C. The CD71 or CD235a positive cells were sorted by LS columns.

Three-germ layer differentiation

iPSCs were respectively differentiated into endoderm, ectoderm, and mesoderm using the STEMdiff™ Trilineage Differentiation kit (STEMCELL Technologies). Each differentiated cell was fixed with BD Cytofix™ Fixation Solution buffer and stained with specific antibodies. For endoderm (SOX17), ectoderm (Nestin) and mesoderm (α-SMA and MIXL1). Nuclei were detected using Hoechst. The data was acquired by Leica fluorescence microscopy. The antibodies used: SOX17 (Novus), Nestin (Abcam), SMA (Sigma-Aldrich) MIXL1 (ProteinTech).

Off-target

Off-target lists were determined as top 10 ranks by online design tool, Benchling (http://www.benchling.com). These sites were amplified by conventional PCR and further confirmed by Sanger sequencing.

Quantitative real-time PCR

Total RNA was extracted by using RiboEX™ (GeneAll Biotechnology) according to the manufacturer's instructions. 1 μg of total RNA converted into cDNA using ReverTra Ace-α-cDNA synthesis kit (TOYOBO). Real-time PCR was performed using SYBR Green Realtime PCR Master Mix (TOYOBO) based on the manufacturer's instructions with primers to detect Orai1, HBA, HBB, KLF1, ALAS2, Band3, EPOR, Protein4.2, TAL1, and DDX5. Real-time PCR was conducted using Light Cycler 480 II (Roche) with the following parameters: Pre-incubation (95 °C, 5 min), 45 cycles of amplification (95 °C, 10 s; 60 °C, 10 s; 72 °C, 10 s), melting curve analysis (95 °C, 5 s; 65 °C, 1 min; 97 °C, continuous), and cooling at 40 °C. Each RNA sample was applied in triplicate, and the relative gene expression level was determined by the comparative threshold cycle (Ct) method. The primer sequence information is provided in Supplementary Table 2.

Immunocytochemistry

CD235a positive cells, differentiated from iPSCs and H9, were fixed and permeabilized with BD Cytofix/Cytoperm™ Fixation and Permeabilization Solution buffer (BD biosciences) for 20 min in RT. It was attached to a slide using Thermo Scientific Shandon 4 Cytospin and stained with antibodies diluted BD 1x perm/wash buffer.

Nuclei were stained with Hoechst. KLF1 (sigma-Aldrich) or beta-globin (Santa Cruz Biotechnology) antibody was detected under Zeiss LSM confocal microscopy.

Cytospin

After fixation with 4% PFA (Thermo Fisher Scientific), the 10^5 HUDEP-2 cells in 200 μl PBS were spun onto slides at 400 rpm for 5 min. Spinning was done by using Thermo Scientific Shandon 4 Cytospin. The slides were air-dried before staining.

Plasmids

For the viral infection, we used the pLTe backbone. As a control, the lentiviral vector pLTe-EGFP (Addgene) was used. To construct the Orai1 R91W expression vector, human Orai1 cDNA was amplified by polymerase chain reaction (PCR) with the following primers: 5'-GGATCC ACCTGCATCCTGCCCAACATCGAG-3' (sense) and 5'- TCAGGCCCAGGCCAGCTCGATGTGGCG-3'(Antisense). The PCR product was subcloned into PCY3 entry vectors using T4 DNA ligase (NEB) according to the manufacturer's protocol. Orai1 R91W mutation was constructed by QC PCR substitution with the primer sets: 5'-CTTAAAGCC TCCAGCTGGACCTCGGCTCTG-3' (Top) and 5'-CAGA GCCGAGGTCCAGCTGG AGGCTTTAAG-3' (Bottom). After confirmation of the DNA sequence, Orai1 R91W cDNA was transferred to the pLTe-DEST-GFP-X plasmid using Gateway LR clonase (Invitrogen).

Lentiviral production and cell infection

To generate lentiviral particles, HEK293T17 cells were transfected with pVSV-G (viral packaging vector), p8.2 (viral genomic vector), and target gene coding lentiviral vectors using jetPRIME (Polyplus) according to the manufacturer's protocols. 36 h after the transfection, the culture medium was spun at 7000 rpm for 10 min at 4 ℃ and treated in HUDEP-2 for 4 h. Infection efficiency was assessed by measuring GFP+ cells after 2 days of infection.

May-Grünwald Giemsa stain

$1X10^5$ of HUDEP-2 cells were spun onto slide glass (Thermo Fisher Scientific) with a Shandon 4 cytospin and air dried for 5min at room temperature. The slide glasses were incubated with May-Grunwald working solution (Sigma-Aldrich) for 3 min and an

additional 1 min with 1x PBS. Next, the samples were treated with 1x Giemsa staining solution (Sigma-Aldrich) for 5 min, followed by washing steps with distilled water. Sample images were examined using an Evos microscope (EVOS XL Core) and a Bioimaging navigator (Olympus). Cells were selected by random sampling, and representative fields were used for qualitative assessment of erythroid maturation.

ChIP assay

5×10^6 of HUDEP-2 cells were used per IP sample. The ChIP protocol was followed according to the instructions included in the ChIP kit (Cell Signaling Technologies). Protein-DNA complexes were formed by incubating the cells within 1 % formaldehyde (Sigma-Aldrich) in growth media for 10min at RT. The complexes were immunoprecipitated with 4 μg anti-NFATc1 (Santa Cruz Biotechnology) antibody overnight. NFAT2 binding to the KLF1 promoter was quantified by real-time qPCR using a SYBR green PCR kit (TOYOBO). Primer pairs of P1, and P2 site of the KLF1 promoter region, were as follows: P1, forward, 5'-CTGTGGAGCCTCAATCAG-3', reverse, 5'-AGACATTGGGTCTCCAAAGAAG-3', P2, forward, 5'-GTGTCACCCAGGTTGATCTG-3', reverse, 5'-GGGACTGCACTGAACTATAAT-3', and P3, forward, 5'-GTGTCACCCAGGTTGATCTG-3', reverse, 5'-GGGACTGCACTGAACTATAAT-3'.

Luciferase assay

1×10^6 of HUDEP-2 cells were electroporated using a gene Pulser Xcell electroporation system (Bio-Rad) with 2 μg of reporter, and 1 μg of the pRLTK control vector. After incubation for 24 hours, the cells were treated with TG (0.1 μM) or CsA (0.1 μM) for 12 h. Luciferase activity was analyzed using the Dual-Luciferase assay system (Promega).

Supplementary Table 1. Primary antibodies for Western blot.

Name	Catalogue #	Company
Orai1	SC-377281	Santa Cruz Biotechnology
β-globin	sc-21757	Santa Cruz Biotechnology
γ-globin	sc-21756	Santa Cruz Biotechnology
KLF1	ab175372	Abcam
NFAT2	SC-7294	Santa Cruz Biotechnology
STAT5	94205T	Cell Signaling Technology
P-STAT5	9359S	Cell Signaling Technology
GFP	598	MBL
Lamin B1	ab16048	Abcam
γ-Tubulin	ab27074	Abcam
β-actin	66009-1-Ig	Proteintech
GAPDH	60004-1-Ig	Proteintech

Supplementary Table 2. Primer sequences for real-time PCR

Name	Forward	Reverse
Orai1	CCCTTCGGCCTGATCTTTATCG	TCCCCTCTGTGGTCCAGCTGGTCC
HBA	GACCCGGTCAACTTCAAGC	AGAAGCCAGGAACTTGTCCA
HBB	GCA CGTGGATCCTGAGAACT	CACTGGTGGGGTGAATTCTT
KLF1	GGTGTGATAGCCGAGAC	GCGTATGGCTTCTCCC
ALAS2	GGAGCGTGATGGAATTATGC	GATTCTAGAGCTCCAGAGAGC
Band3	AATCTGGAGCAGGAGGAATATG	TGATGTGGTGTGGTAGTCTGTG
EPOR	GAGCATGCCCAGGATACCTA	CATGGCCACTATGTCCACAC
Protein4.2	ACCCAAGTGCTCCTAATGGAGG	CCATCCTCACAGCACTTCCAGA
TAL1	TTGTGCGGCGTATCTTC	CAGGGTCCTTGCCAGTC
DDX5	AGAGAGGCGATGGGCCTATTT	CTTCAAGCGACATGCTCTACAA

Supplementary Figure 1. Orai1 Ca^{2+} channel decreases as erythroblasts mature.

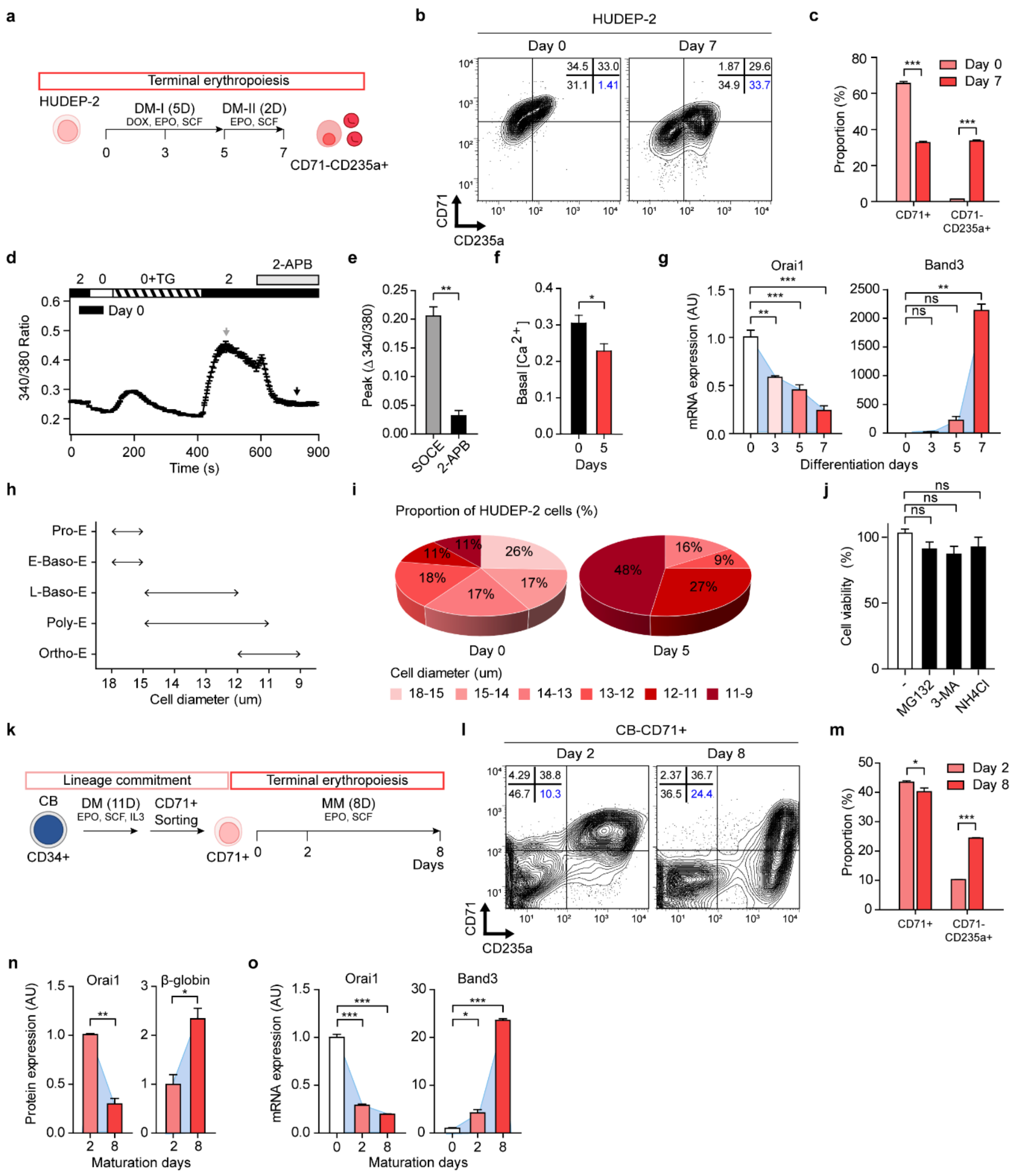

a Schematic representation of the differentiation protocol of HUDEP-2. DOX: Doxycycline, EPO: erythropoietin, and SCF: stem cell factor. **b** Representative FACS analysis of CD71 and CD235a of HUDEP-2 cells following maturation for 7 days. **c** Proportion of $CD71^{+}$, and $CD71^{-}$ $CD235a^{+}$ in HUDEP-2 cells maturated for 7 days (n=3). **d** Fura-2 Ca^{2+} imaging results of HUDEP-2 cells in maturation day 0. **e** Comparison of average Ca^{2+} peak level shown in Fig s1d. The analyzed time point was indicated with a gray arrow (after TG induction), and a black

arrow (after 2-APB injection) (n=65). **f** Comparison of average basal Ca^{2+} level shown in Fig 1a (n≥50). **g** RT-qPCR results of Orai1, and Band3 during maturation of HUDEP-2 (n=3). **h** A pie chart showing the cell size distribution of HUDEP-2 cells. The proportion of cells within 18-15, 15-14, 14-13, 13-12, 12-11, and 11-9 µm were distributed for unmatured (Day 0) and maturated state (Day 5). **i** A graph showing the erythroblast cell type distribution according to the cell diameter. **j** Cell viability after 1 day of maturation. Histograms represent the percentage, concerning control cells (100%) (n=3). **k** Schematic representation of the differentiation protocol of CB-derived $CD71^+$ erythroblasts. EPO: erythropoietin, SCF: stem cell factor, and IL3: interleukin 3. **l** Representative FACS analysis of CD71 and CD235a of CB-derived erythroblasts following maturation for 2 and 8 days. **m** Proportion of $CD71^+$, and $CD71^-$ $CD235a^+$ in CB-derived erythroblast cells maturated for 2 and 8 days (n=3). **n** The densitometric quantification graph of western blot bands of Fig. 1i (n=3). **o** RT-qPCR results of Orai1, and Band3 during maturation of CB-$CD71^+$ cells (n=3). Data are mean ±SEM. p values were calculated using an unpaired two-tailed t-test (c, e, f, m, n), and one-way ANOVA Dunnett's post-hoc test (g, j, o) (ns, $p > 0.05$; *$p \le 0.05$; **$p \le 0.01$; ***$p \le 0.001$).

Supplementary Figure 2. Orai1 inactivation enhances terminal maturation of HUDEP-2.

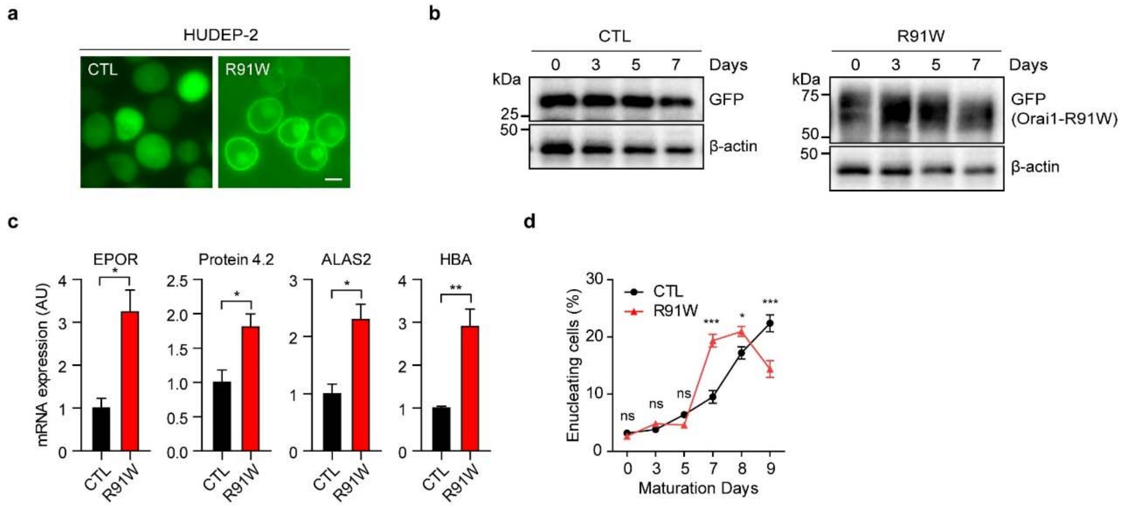

a Fluorescence images of GFP CTL and R91W mutants. Scale bar=10 μm. **b** Protein expression analysis by western blotting of the two HUDEP-2 cell lines expressing GFP and GFP-Orai1 R91W on maturation days 0, 3, 5, and 7. **c** RT-qPCR results of terminal erythroid marker genes (EPOR, Protein4.2, ALAS2, and HBA) in CTL and R91W mutants on maturation day 3. **d** Proportion of enucleating cells which is depicted in Figure 3k. Data are mean ±SEM. p values were calculated using an unpaired two-tailed t-test. Statistical analysis in **d** was performed using two-way ANOVA with Bonferroni posttests (ns, $p > 0.05$; *$p \le 0.05$; **$p \le 0.01$; ***$p \le 0.001$).

Supplementary Figure 3. Orai1 activation inhibits terminal maturation of HUDEP-2.

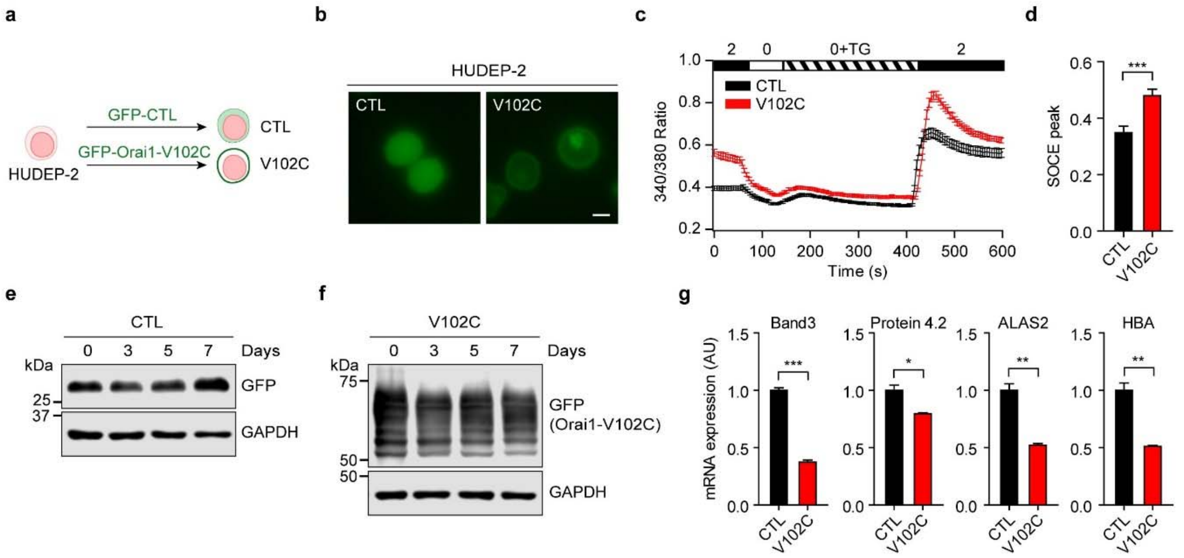

a Schematic representation of method constructing N-terminally GFP-tagged Orai1 V102C and GFP control over-expressed HUDEP-2 cells. **b** Fluorescence images of GFP CTL and V102C mutants. Scale bar=10 μm. **c** Fura-2 Ca^{2+} imaging results of GFP control (black) and Orai1 V102C (red) HUDEP-2 cells. **d** Comparison of the average SOCE peak level in Supplementary Fig. 3c (n=70). **e-f** Protein expression analysis by western blotting of the two HUDEP-2 cell lines expressing GFP and GFP-Orai1 V102C on maturation days 0, 3, 5, and 7. **g** RT-qPCR results of terminal erythroid marker genes (Band3, Protein4.2, ALAS2, and HBA) in CTL and V102C mutants on maturation day 3. Data are mean ±SEM. p values were calculated using an unpaired two-tailed t-test. (ns, $p > 0.05$; $*p \leq 0.05$; $**p \leq 0.01$; $***p \leq 0.001$).

Supplementary Figure 4. Orai1 decreases as hPSC-derived CD71⁺ erythroblasts mature.

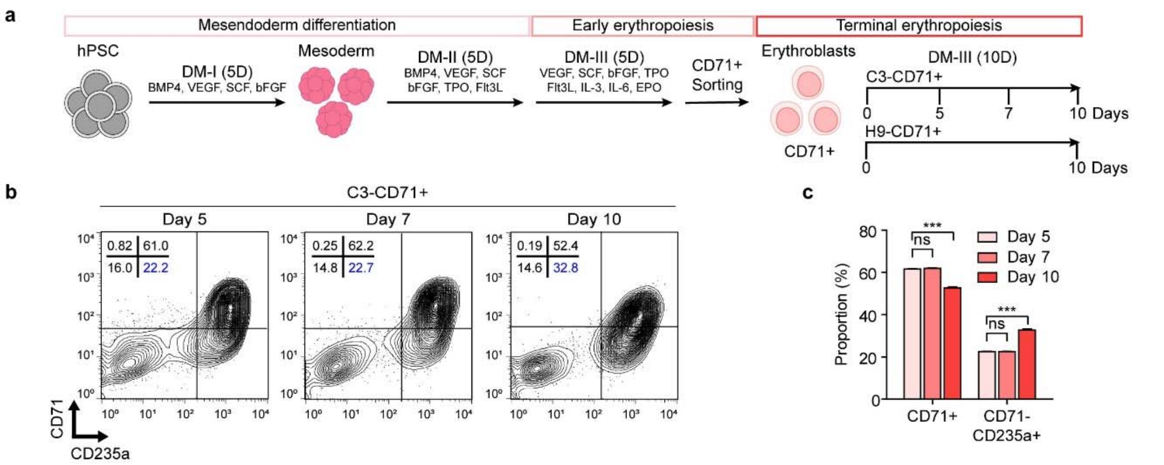

a Schematic representation of the maturation protocol of hPSC-derived CD71⁺ erythroblasts. BMP4: bone morphogenetic protein 4, VEGF: vascular endothelial growth factor, bFGF: basic fibroblast growth factor, TPO: Thrombopoietin, Flt3L: flt-3 ligand, and IL6: interleukin-6. **b** Representative FACS analysis of CD71 and CD235a of C3-derived erythroblast cells following maturation for 5, 7, and 10 days. **c** Proportion of CD71⁺, and CD71⁻CD235a⁺ in C3-erythroblasts maturated for 5, 7, and 10 days. Data are mean ±SEM. p values were calculated using one-way ANOVA Dunnett's post-hoc test (ns, $p > 0.05$; *$p \le 0.05$; **$p \le 0.01$; ***$p \le 0.001$).

Supplementary Figure 5. Establishment of Orai1 KO hPSCs.

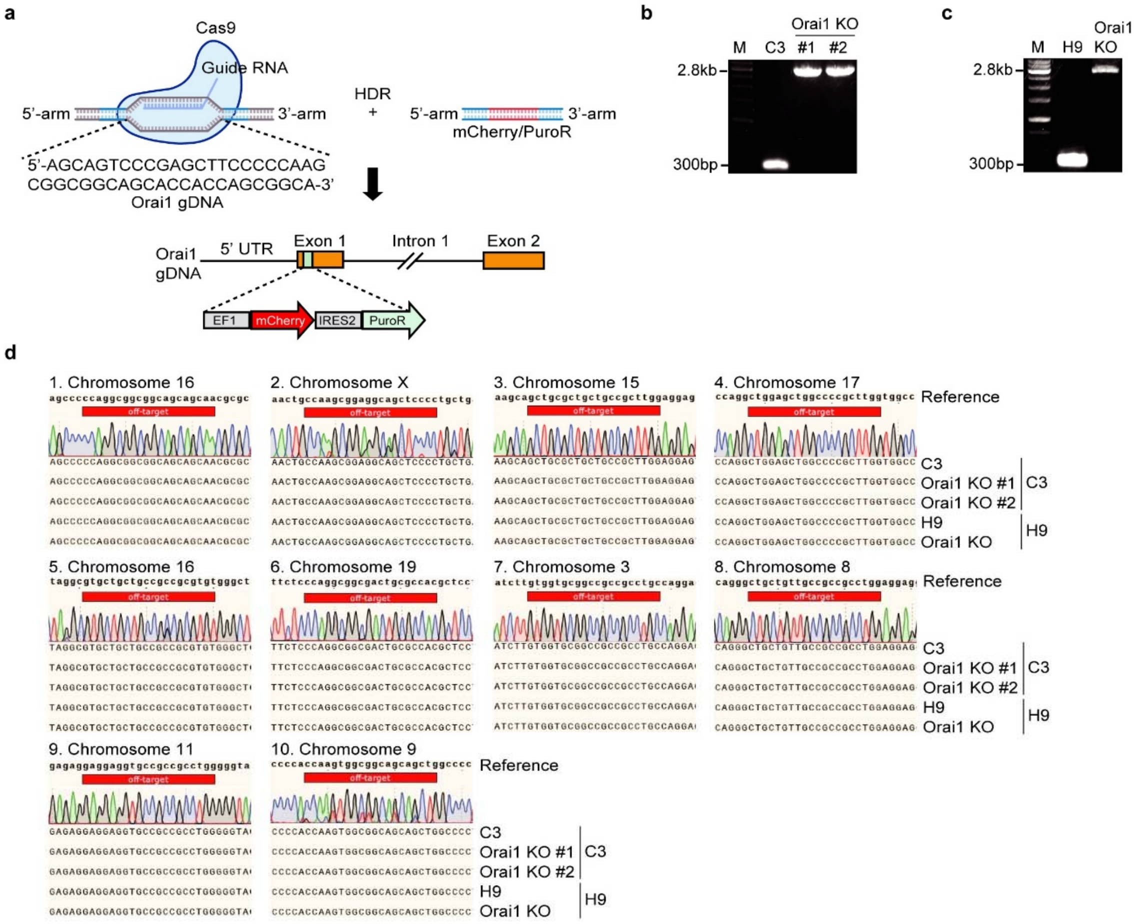

a Schematic diagram of the establishment of Orai1 KO hPSCs using CRISPR-Cas9. **b-c** PCR results of gene insertion in Orai1 KO C3 (b) and H9 (c) cells. **d** Top 10 expected off-target sites of Orai1 sgRNA in the human genome (top). Genomic DNA sequencing results of expected off-target sites from WT and Orai1 KO hPSCs (btm).

Supplementary Figure 6. Orai1 inactivation does not affect pluripotency and three germ layers differentiation of hPSC.

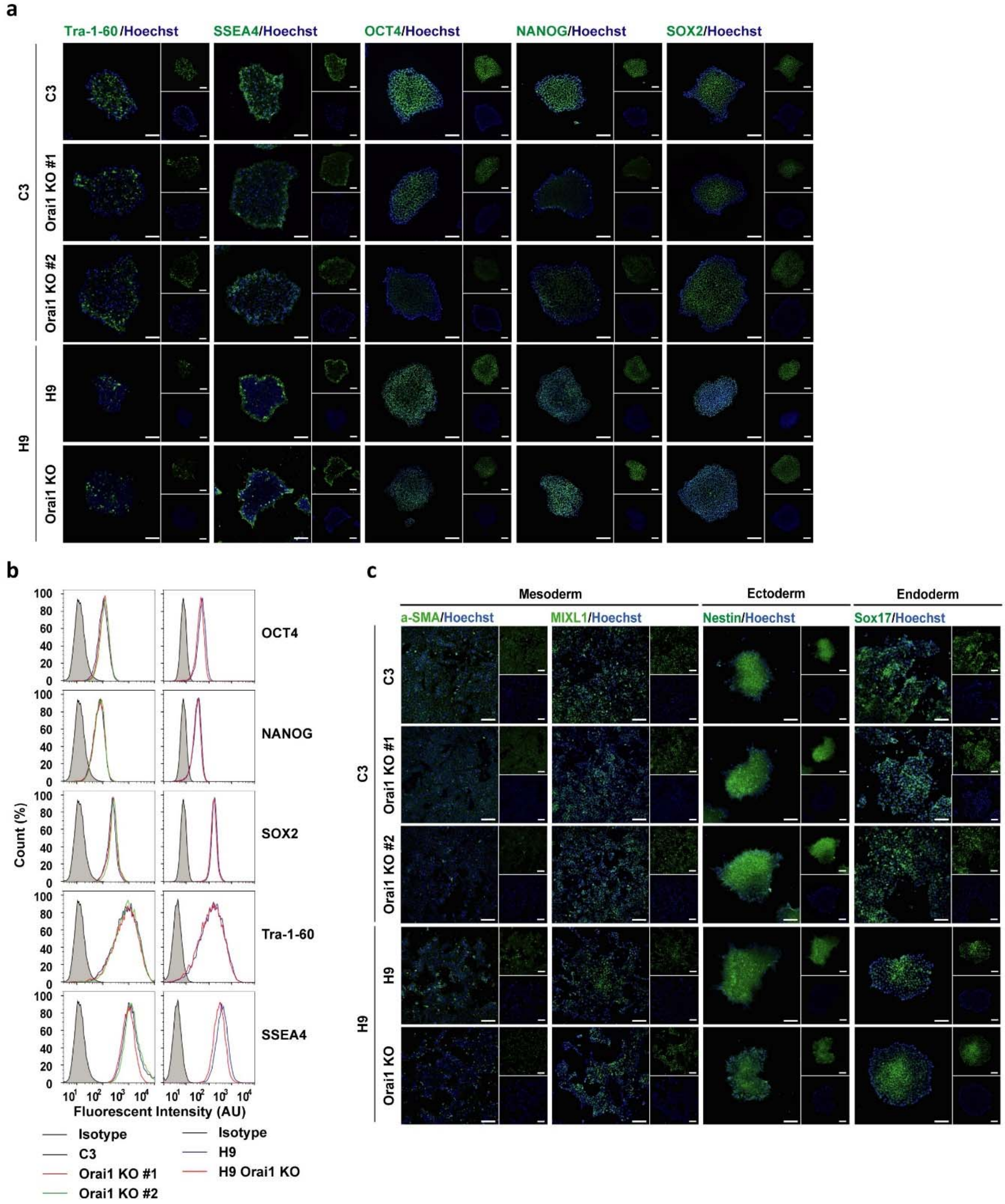

a Immuno-fluorescent images showing expression of surface antigen, Tra-1-60, and SSEA4 as well as transcription factors OCT4, Nanog, and Sox2 in WT versus Orai1 KO hPSCs. Scale bar=100 μm. **b** FACS analysis showing expression of OCT4, Nanog, Sox2, Tra-1-60, and SSEA4 in WT versus Orai1 KO hPSCs. **c** Immuno-fluorescent images showing marker protein

of mesoderm (SMA and MIXL1), ectoderm (Nestin), and endoderm (Sox17) in WT versus Orai1 KO hPSCs. Scale bar=100 μm.

Supplementary Figure 7. Inactivation of Orai1 enhances terminal maturation of iPSC-derived erythroblasts.

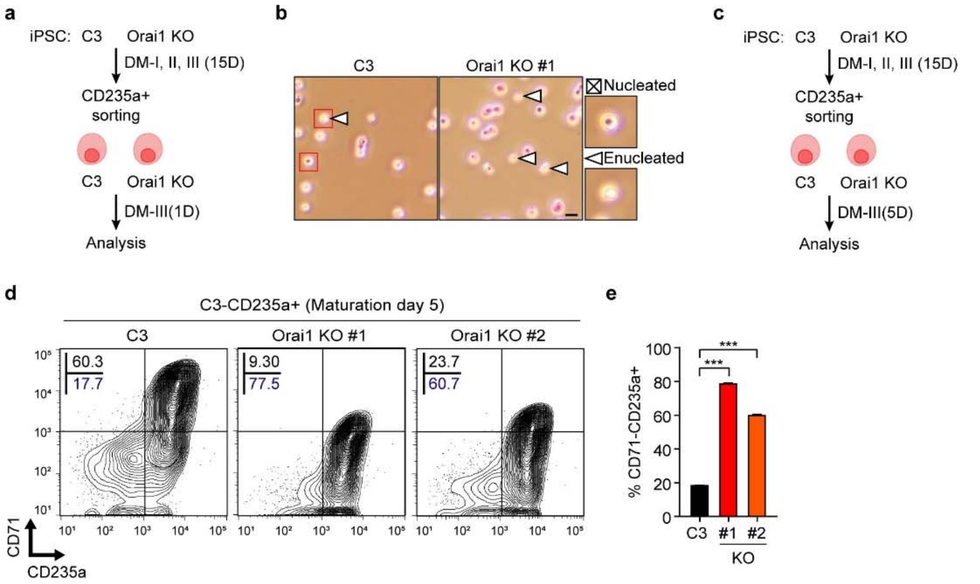

a Schematic diagram of the experimental protocol for WT and Orai1 KO C3-derived CD235a+ cells after maturation for 1 day. **b** May Grunwald Giemsa staining of WT and Orai1 KO cells. Enucleated cells are indicated with a white arrowhead and nucleated cells are not indicated with any symbol. Scale bar=10 μm. **c** Schematic diagram of the experimental protocol for WT and Orai1 KO C3-derived $CD235a^{+}$ cells after maturation for 5 days. **d** Representative FACS analysis of CD71 and CD235a in WT and Orai1 KO C3 following differentiation for 5 days. **e** The proportion of $CD71^{-}CD235a^{+}$ cells in WT and Orai1 KO C3 derived erythroblasts (n=3). Data are mean ±SEM. p values were calculated using one-way ANOVA with Bonferroni's post-hoc test (ns, p > 0.05; *p ≤ 0.05; **p ≤ 0.01; ***p ≤ 0.001).

Supplementary Figure 8. Inactivation of Orai1 enhances terminal maturation of H9-derived erythroblasts.

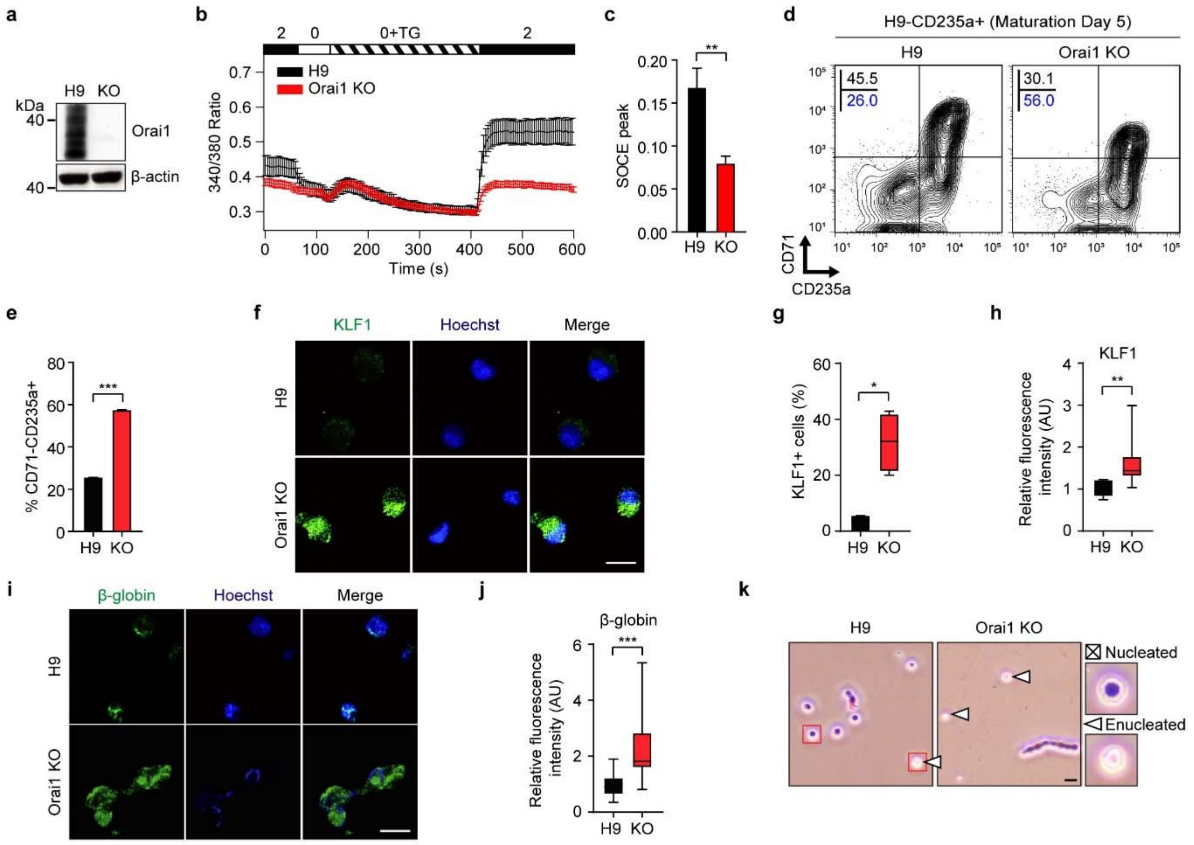

a Representative western blot showing Orai1 protein in WT and Orai1 KO H9. **b** Fura-2 Ca^{2+} imaging results of WT (black) and Orai1 KO (red) H9. **c** Comparison of average SOCE peak level of WT and Orai1 KO H9. **d** Representative FACS analysis of CD71 and CD235a in WT and Orai1 KO H9-CD235a^{+} cells following differentiation for 5 days. **e** The proportion of CD71^{-} CD235a^{+} cells in WT and Orai1 KO H9 derived erythroblasts (n=3). **f** Immuno-fluorescent images showing KLF1 expression in WT and Orai1 KO iPSC-CD235a^{+} cells. Scale bar=20 μm. **g** The proportion of KLF1-positive cells in WT versus Orai1 KO cells. **h** Relative fluorescence intensity of endogenous KLF1 in WT versus Orai1 KO cells. **i** Immuno-fluorescent images showing β-globin expression in WT and Orai1 KO cells. Scale bar=20 μm. **j** Relative fluorescence intensity of endogenous β-globin in WT versus Orai1 KO cells. **k** May Grunwald Giemsa staining of WT and Orai1 KO cells. Enucleated cells are indicated with a white arrowhead and nucleated cells are not indicated with any symbol. Scale bar=10 μm. Data are mean ±SEM. p values were calculated using an unpaired two-tailed t-test (ns, $p > 0.05$; *$p \le 0.05$; **$p \le 0.01$; ***$p \le 0.001$).

Supplementary Figure 9. Orai1 activation inhibits KLF1 expression.

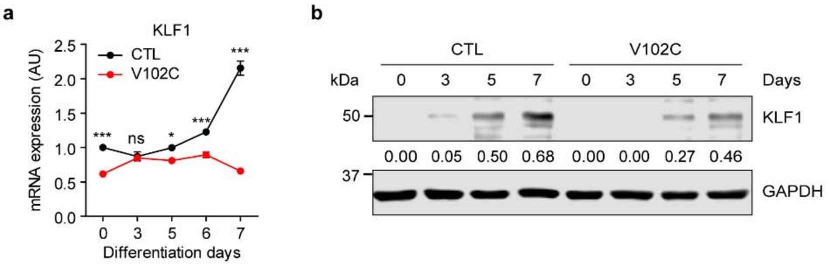

a RT-qPCR results of KLF1 gene in CTL and V102C mutants on maturation day 0, 3, 5, 6, 7. **b** KLF1 protein expression analysis by western blotting of the two HUDEP-2 cell lines expressing GFP and GFP-Orai1 V102C on maturation days 0, 3, 5, and 7. Data are mean ±SEM. p values were calculated using two-way ANOVA with Bonferroni posttests (ns, $p > 0.05$; *$p \le 0.05$; **$p \le 0.01$; ***$p \le 0.001$).

Supplementary Figure 10. Orai1-mediated NFAT2 inhibits KLF1 transcription.

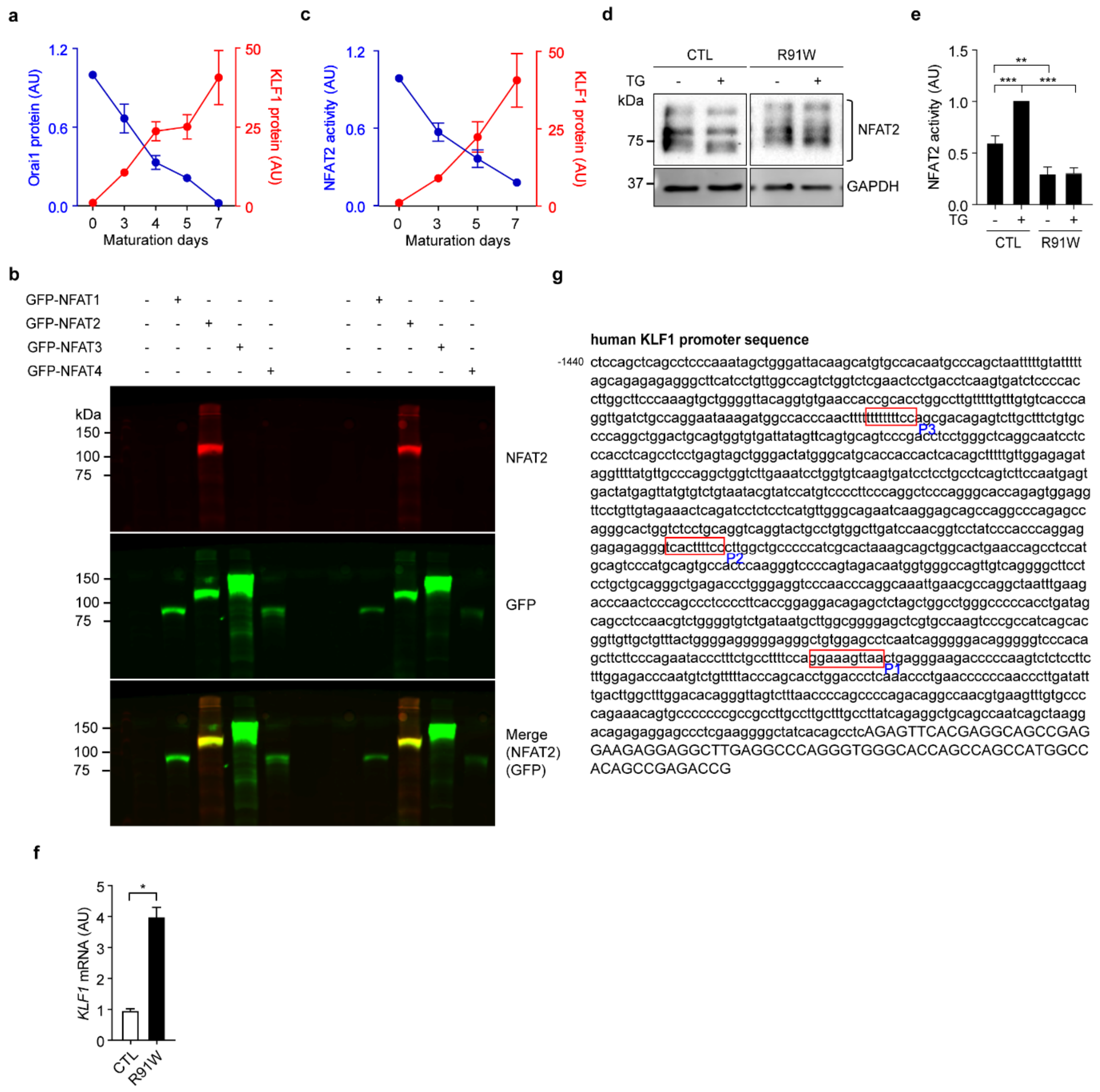

a The densitometric quantification graph of western blot bands which is shown in Figure 5b. **b** Western blot analysis of GFP-NFAT1, 2, 3, and 4 overexpressed HEK293T cells. **c** Relative expression of KLF1 protein was compared with NFAT2 activity during terminal erythropoiesis. **d** Western blot analysis of NFAT2 in GFP CTL and Orai1 R91W mutant cells under 1 μM TG treatment. **e** Graph plots relative NFAT2 activity (n=3). **f** Relative mRNA expression level of KLF1 of GFP CTL and Orai1 R91W mutant cells on differentiation day 3. **g** The Nucleotide sequence of 1,440 bp DNA element upstream of the transcription start site (TSS) containing the KLF1 gene promoter. The three putative NFAT2 binding sites are indicated in the red box. Data is mean ±SEM. p values were calculated using one-way ANOVA with Bonferroni's post-hoc test (e), and unpaired two-tailed t-test (f) (ns, $p > 0.05$; *$p \leq 0.05$; **$p \leq 0.01$; ***$p \leq 0.001$).

Supplementary Figure 11. EPO orchestrates dual mechanisms of KLF1 transcription, encompassing Orai1-NFAT2 downregulation followed by STAT5 maintenance.

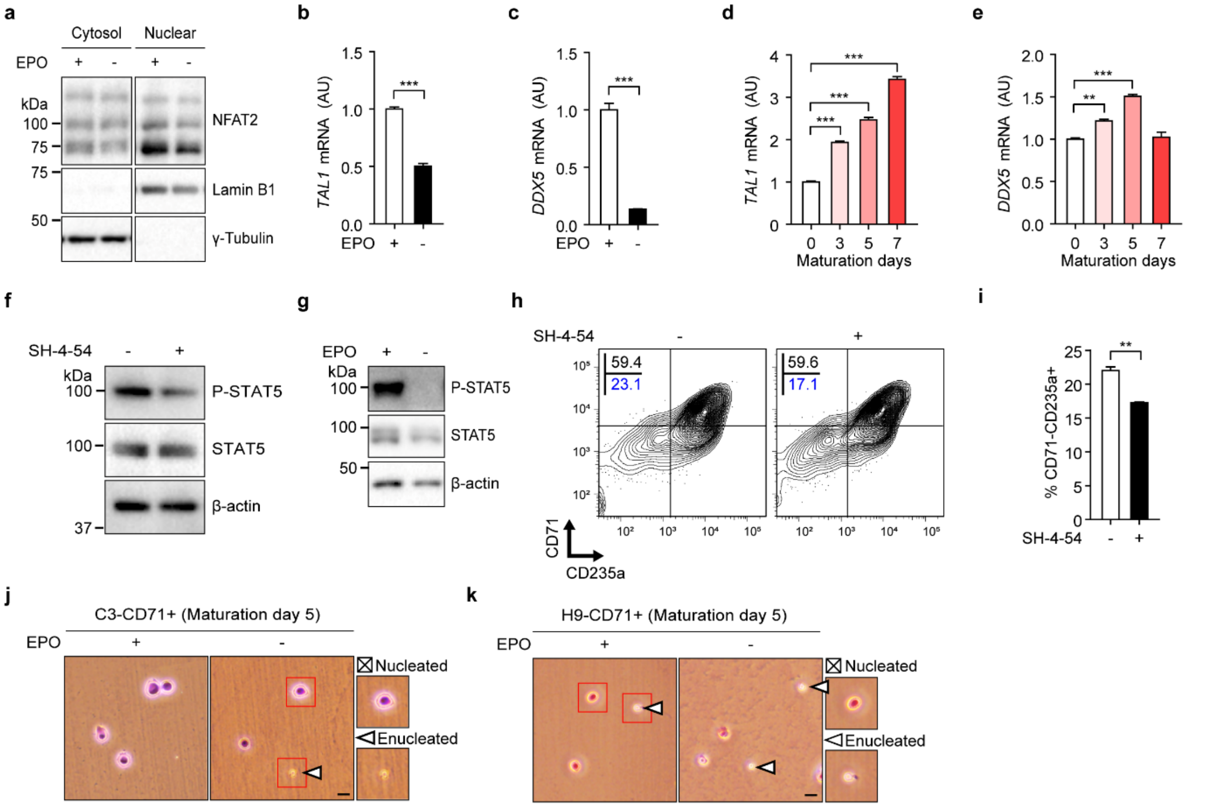

a Immunoblot analysis of cytoplasmic and nuclear NFAT2 in HUDEP-2 with or without EPO. Anti-tubulin antibody was used as loading control for the cytoplasmic fraction and anti-LaminB1 antibody for the nuclear fraction. **b-c** Relative mRNA expression level of TAL1 (b) and DDX5 (c) with or without EPO from day 5 to 7 of differentiation. **d-e** Relative mRNA expression of TAL1 (d) and DDX5 (e) in maturation day 0, 3, 5, and 7. **f** Representative blot image of P-STAT5, STAT5, and β-actin expression in HUDEP-2 with or without SH-4-54. **g** Western blot analysis of P-STAT5, STAT5, and β-actin in HUDEP-2 with or without EPO from day 5 to 7 of differentiation. **h** Representative FACS analysis of CD71 and CD235a of HUDEP-2 with or without SH-4-54. **i** The proportion of $CD71^-CD235a^+$ cells in normal versus SH-4-54 treated conditions. **j-k** May Grunwald Giemsa staining of C3-derived (j) and H9-derived (k) $CD71^+$ cells in EPO-deprived (right) or normal (left) condition after 5-day maturation. Data are mean ±SEM. p values were calculated using an unpaired two-tailed t-test (b, c, i) and one-way ANOVA Dunnett's post-hoc test (d, e) (ns, $p > 0.05$; *$p \leq 0.05$; **$p \leq 0.01$; ***$p \leq 0.001$).
